# High and fast: NMR protein–proton side-chain assignments at 160 kHz and 1.2 GHz†

**DOI:** 10.1039/d3sc03539e

**Published:** 2023-09-20

**Authors:** Morgane Callon, Dominique Luder, Alexander A. Malär, Thomas Wiegand, Václav Římal, Lauriane Lecoq, Anja Böckmann, Ago Samoson, Beat H. Meier

**Affiliations:** a Physical Chemistry, ETH Zürich 8093 Zürich Switzerland morgane.callon@phys.chem.ethz.ch beme@ethz.ch; b Molecular Microbiology and Structural Biochemistry (MMSB) UMR 5086, CNRS, Université de Lyon, Labex Ecofect 7 passage du Vercors 69367 Lyon France; c Institute of Cybernetics, Spin Design Laboratory, Tallinn University of Technology Tallinn Estonia

## Abstract

The NMR spectra of side-chain protons in proteins provide important information, not only about their structure and dynamics, but also about the mechanisms that regulate interactions between macromolecules. However, in the solid-state, these resonances are particularly difficult to resolve, even in relatively small proteins. We show that magic-angle-spinning (MAS) frequencies of 160 kHz, combined with a high magnetic field of 1200 MHz proton Larmor frequency, significantly improve their spectral resolution. We investigate in detail the gain for MAS frequencies between 110 and 160 kHz MAS for a model sample as well as for the hepatitis B viral capsid assembled from 120 core-protein (Cp) dimers. For both systems, we found a significantly improved spectral resolution of the side-chain region in the ^1^H–^13^C 2D spectra. The combination of 160 kHz MAS frequency with a magnetic field of 1200 MHz, allowed us to assign 61% of the aliphatic protons of Cp. The side-chain proton assignment opens up new possibilities for structural studies and further characterization of protein–protein or protein–nucleic acid interactions.

## Abstract 2

The combination of the fastest MAS and highest magnetic field allows to spectrally resolve the side-chain protons of proteins. It makes ^1^H-detected MAS NMR a key player in the study of protein interactions with other macromolecules or nucleic acids.

## Graphical abstract

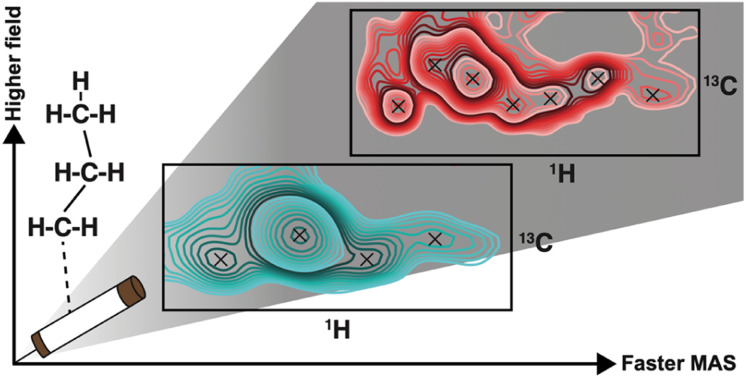

## Introduction

Fast magic-angle spinning (MAS) at rotational frequencies *ν*_r_ around 100 kHz, combined with proton detection (^1^H-MAS-NMR), has recently brought significant improvements in resolution and sensitivity^1,2^ to solid-state NMR spectroscopy. In addition, the increase in mass sensitivity by a factor of about 50 thanks to proton detection at fast MAS with rotors of 0.7 mm diameter compared to carbon detection in 3.2 mm ones,^3^ now allows the study of complex proteins that are only available in small amounts.^4–6^ The best spectral resolution is usually observed for the backbone amide (H^N^) and the H_α_ protons while the side-chain protons are typically not well-resolved even at the highest available magnetic fields.^7^ They feature a small chemical-shift dispersion. Therefore, most dipole-coupled protons are in an intermediate or strong coupling regime,^7,8^ where the homonuclear dipolar line broadening, as described by the higher order average Hamiltonian terms, is at least comparable to the chemical-shift separation at the magnetic fields currently used for protein NMR. This induces spectral overlap of these resonances. They are however crucial notably for structure determination, since, especially in the hydrophobic core, they form most of the essential contacts that define the protein fold. Proton spectra can also be important for the characterization of biomolecular interactions, as protons are localized at protein–protein interfaces, and can thus provide information on dimer contacts in multimers, or between a protein and a ligand or nucleic acid. Resolving these resonances requires a further improvement in spectral resolution, which can be achieved either by reducing their spectral line width and/or increasing the chemical-shift dispersion in the NMR spectra. Selective isotopic dilution of side-chain protons has been developed, and partial labelling of side-chains, particularly methyl groups, has enabled the recording of side-chain ^1^H–^1^H distances by solid-state NMR.^9–15^ In these studies, however, side-chain protons were not systematically assigned, and often methyl groups were exclusively used. Indeed, while methyl groups play an important role in defining the hydrophobic core of proteins, residues not carrying such protons are equally involved in protein–protein interactions, such as aromatic residues involved in π stacking, polar residues in side-chain hydrogen bonds, or cation–π interactions.^16^ Thus, working on fully protonated proteins will open up for investigations of a greater variety of structural interactions, and first attempts in this direction have recently been made on model systems^17–20^ as well as fully protonated membrane proteins^21–26^ at MAS frequencies of 100–111 kHz.

In solid-state NMR spectroscopy, the proton line width consists of a homogeneous contribution, an inhomogeneous one and a chemical exchange part: *Δ*^tot^ = *Δ*^homo^ + *Δ*^inhomo^ + *Δ*^exch^. In the following we assume that the possible contribution from chemical exchange^27^ is negligible here. We also neglect the contribution of ^1^H–^1^H J-couplings which are typically below 15 Hz but can give rise to a (non-resolved) multiplet pattern that contributes to the linewidth.

The inhomogeneous line width is due to magnetic-field heterogeneity and inherent sample heterogeneities, and is largely independent of the spinning frequency, but increases at higher magnetic fields due to the higher chemical shift dispersion. The homogeneous line width consists of a coherent and an incoherent part (*Δ*^homo^ = *Δ*^coh^ + *Δ*^incoh^, assuming Lorentzian lines). The incoherent part is due to transverse relaxation caused by protein dynamics or to chemical exchange, and is acting, albeit differently, both in solution and in solid-state NMR experiments. The coherent contribution, on the other hand, is specific to solid-state NMR, and arises from the anisotropic interactions (dipolar coupling and cross terms with chemical shift anisotropy, CSA) that are not fully averaged by MAS at finite MAS frequencies, as predicted by average Hamiltonian theory (AHT).^28–30^ Thus, in order to reduce the homogeneous line width to become similar to the “natural” line width given by the transverse relaxation, one must decrease the coherent contribution. This contribution was experimentally found to decrease with the inverse spinning frequency in a polynomial manner, containing a linear and a quadratic contribution.^31–33^ For the amide protons of a fully protonated protein, a 19% reduction of the line width was shown when spinning 26% faster (from 100 to 126 kHz MAS), and a further reduction by 25% when increasing the spinning frequency to 150 kHz.^31,33^ The side-chain protons, which are in a relatively strong coupling regime, exhibit a larger homogeneous line width at a given spinning frequency than H_α_ or H^N^. Higher magnetic field has the potential to further reduce their line width by increasing the chemical-shift differences leading to a weaker coupling regime.^7^ We show how the combination of high magnetic field, at 1200 MHz proton Larmor frequency, and fast MAS at 160 kHz frequency, positively affects the proton line widths, and allows proton side-chain resonance assignments of the HBV capsid core protein (Cp149). We first evaluate this gain in resolution on a model system, the phosphorylated amino acid *O*-phospho-l-serine. Then, we investigate the line widths of the side-chain resonances of Cp149. While the ^1^H^N^,^13^C,^15^N assignments have been determined previously,^34^ we assigned, combining fast spinning at 160 kHz and high magnetic field, 61% of the H_α_ and side-chain proton resonances using three-dimensional ^1^H-detected spectra.

## Material and methods

### Sample preparation

*O*-Phospho-l-serine has been purchased from Sigma Aldrich and was used without further processing. The sample is fully protonated and contains predominantly ^14^N and ^12^C. Protonated and uniformly [^13^C–^15^N]-labeled (UL) Cp149 empty capsids (not containing nucleic acids) were produced and purified as described in Lecoq *et al.*^34^ In brief, Cp149 protein was expressed in *E. coli* BL21(DE3)*CP strain, grown at 37 °C in LB medium containing 1 g L^−1^ of ^15^NH_4_Cl and 2 g L^−1^ of ^13^C-glucose in H_2_O and supplemented with 100 μg mL^−1^ ampicillin and 34 μg mL^−1^ chloramphenicol. When the OD_600_ reached 0.7, the expression was induced with 1 mM IPTG and the culture was further incubated for 6 h at 25 °C. Harvested cells were resuspended in 15 mL of lysis buffer (50 mM Tris pH 7.5, 300 mM NaCl, 5 mM DTT) and incubated on ice for 45 min with 1 mg mL^−1^ of chicken lysozyme, 1× of protease inhibitor cocktail solution and 0.5% of Triton-X-100, then mixed with 4 μL of Benzonase nuclease for 30 min at room temperature. Cells were broken by sonication and centrifuged for 1 h at 8000*g* to remove cell debris. UL-Cp149 capsids in the supernatant were purified on a stepwise sucrose gradient from 10 to 60% (m/v) sucrose (buffered in 50 mM Tris pH 7.5, 300 mM NaCl) which was centrifuged at 28 800*g* for 3 h at 4 °C. The fractions containing UL-Cp149 were further purified by (NH_4_)_2_SO_4_ precipitation (up to 35% saturation) and finally dialyzed in the solid-state-NMR buffer (50 mM Tris pH 7.5, 5 mM DTT) overnight at 4 °C. 20 μL of saturated 4,4-dimethyl-4-silapentane-1-sulfonic acid (DSS) solution were added to the protein for chemical-shift referencing prior to the sedimentation step. Sub-milligram amounts of capsids were filled into a 0.5 mm rotor using home-made filling tools^35^ by centrifugation (200 000*g*, 17 h, 4 °C).

### NMR spectroscopy, data processing and analysis

Solid-state NMR experiments were recorded at static magnetic-field strengths of 20.0 and 28.2 T (wide-bore 850 MHz Bruker Avance III and standard-bore 1200 MHz Bruker Avance NEO spectrometer, respectively). The spectra of *O*-phospho-L-ser were acquired using a 0.5 mm triple-resonance probe head (Ago Samoson, Darklands OÜ, Tallinn, Estonia). For the resonance-specific investigation of the homogeneous line width at 850 and 1200 MHz proton frequency, sixteen and eighteen 2D-hCH 
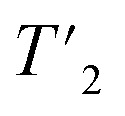
 relaxation experiments with echo-delay time varied from 0.002 to 6 ms and 0.001 to 10 ms respectively were measured. No cooling was applied during the measurement of the *O*-phospho-l-ser spectra, thus the sample temperature increased with increasing MAS frequency according to the Fig. S1.†

Solid-state NMR spectra of UL-Cp149 were recorded on capsids sedimented^36,37^ in H_2_O and internally referenced to DSS. The spectra were acquired using 0.5 mm triple-resonance probe heads and spinning frequencies of 110 and 160 kHz MAS. The sample temperature of around 25 °C was determined from the relation *T* (°C) = 455 − 90*δ*_H_2_O_ where *δ*_H_2_O_ denotes the supernatant water chemical shift in ppm.^35^ The sample temperature was kept constant at 25 °C at all MAS frequencies by using an air-cooling system which compensates the MAS sample heating due to fast sample rotation shown in Fig. S1.†

The 2D-hCH experiments were recorded with 800 μs cross-polarization (CP) transfers. For the residue-specific measurement of the homogeneous line width, eight 2D-hCH 
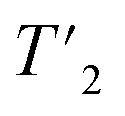
 relaxation experiments with echo-delay times up to 4.5 ms were measured at 110 and 160 kHz MAS frequency and 1200 MHz proton frequency. Bulk proton longitudinal relaxation times *T*_1_ (^1^H) have been determined using a saturation recovery sequence with 16 variable delays up to 7 and 8 s for the H^N^/aliphatic protons (^1^H^ali^) respectively. Bulk proton transverse relaxation times 
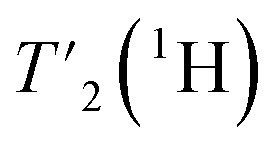
 have been determined using a Hahn-echo sequence with 16 variable echo delays *τ*_echo_ from 1 μs to 6/7 ms for the H^N^/^1^H^ali^ respectively. Bulk proton relaxation times under spin lock *T*_1ρ_(^1^H) have been determined with 10 variable spin lock times from 1 μs to 40 ms (^1^H spin lock rf-field strength of 13 kHz). Bulk ^15^N transverse relaxation times 
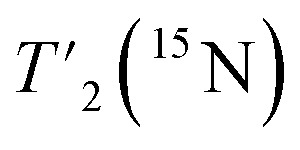
 have been determined using a Hahn-echo sequence with 16 variable echo delays *τ*_echo_ from 1 μs to 35 ms. The 3D-hNCH experiment was recorded with 20 ms Cα-N cross-polarization (CP) transfers. The 3D-hCCH TOBSY experiment used a C9_57_ block^38^ for the TOBSY mixing of 15 ms (see puse program in Fig. S2†) and was recorded using the simultaneous acquisition of a frequency reference (SAFR).^39,44^ As well, additional 3D-hCCH and 3D-HcCH TOBSY experiments were recorded using a WALTZ-16 bloc^40^ for the TOBSY transfer with 30 kHz rf amplitude and with a length of 11 ms. The inter-scan delay for the experiments has been set to the value of 1.27*T*_1_(^1^H^ali^) based on the measured bulk protein *T*_1_(^1^H^ali^) relaxation time. All experiments use the MISSISSIPPI scheme^41^ for solvent suppression, WALTZ-64 heteronuclear decoupling on ^13^C during ^1^H acquisition^40,42^ and frequency-sweep low-power TPPM decoupling on ^1^H during indirect time increments in 2D and 3D experiments.^43^ Detailed information about all acquisition parameters can be found in Tables S1–S4.†

The spectra were processed using Topspin 4.0.6 (Bruker Biospin). The *O*-phospho-l-serine spectra were processed without apodization function and calibrated using an external calibration on the ^13^C spectra of solid adamantane recorded in the same probe head directly after the measurement. The UL-Cp149 spectra were processed with zero filling to the double amount of data points and a shifted sine-bell apodization function in direct and indirect dimensions with SSB = 2.5. Pre-existing ^13^C resonance assignments were transferred from Lecoq *et al.*^34^ (BMRB accession number 27317). The spectra were analyzed in CcpNmr Analysis 2.4.2.^44,45^

### Relaxation and line width analysis

For *O*-phospho-l-serine, site-specific 
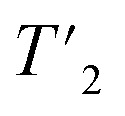
(^1^H) relaxation times were extracted from a series of 1D ^1^H spin-echo experiments, where the peak areas have been determined using the relaxation analysis tool in Topspin and exported to MATLAB (version 9.6.0). Relaxation curves were fitted with a mono-exponential decay function with two free parameters and functional form *A* exp(−*τ*_echo_/*t*) and are shown in Fig. S3 and S4.† For UL-Cp149, site-specific 
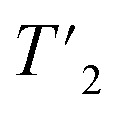
(^1^H) relaxation times were extracted from a series of 2D-hCH 
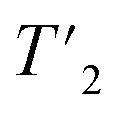
 relaxation experiments, using the spectral fitting package INFOS.^46^ Relaxation curves were fitted with a mono-exponential fit and are shown for all resonances in Fig. S5 and S6.† Site-specific homogeneous line widths have been determined using the relation 
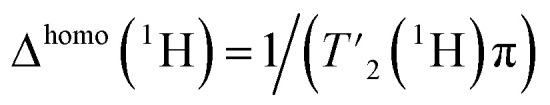
. Experimental errors *σ* have been determined using bootstrapping methods with 200 iterations for relaxation time data and subsequent Gaussian error propagation for the error on the corresponding homogeneous line widths. For *O*-phospho-l-serine, the MAS dependence of the homogeneous line widths *Δ*^homo^(^1^H) were fitted by a quadratic polynomial model of the form *Δ*^homo^(*ν*_r_) = *c*^(1)^ + *c*^(2)^/*ν*_r_ + *c*^(3)^/*ν*_r_^2^, with the spinning frequency *ν*_r_, forcing the *y*-axis intercept to go through the origin (*c*^(1)^ = 0). For UL-Cp149 bulk relaxation measurement, relaxation times were extracted from a series of 1D hcH and hnH experiments, where the peak areas have been determined using the relaxation analysis tool in Topspin and exported to MATLAB. Relaxation curves were fitted with a mono-exponential decay function with two free parameters and functional form *A* exp(−*τ*_echo_/*t*) and are shown in Fig. S7.† For UL-Cp149, site-specific total line widths were extracted from series of 2D-hCH CP-based spectra using the spectral fitting package INFOS.^46^ For *O*-phospho-l-serine, the site-specific total line widths were determined using the line-shape integrated tool in Topspin (Bruker). The 1D spectra are fitted using a mixed Lorentzian and Gaussian function and the full-widths at half maximum (FWHM) corresponding to the total line width is extracted.

## Results

### Line width gain at higher field and faster MAS

**Fig. 1:**
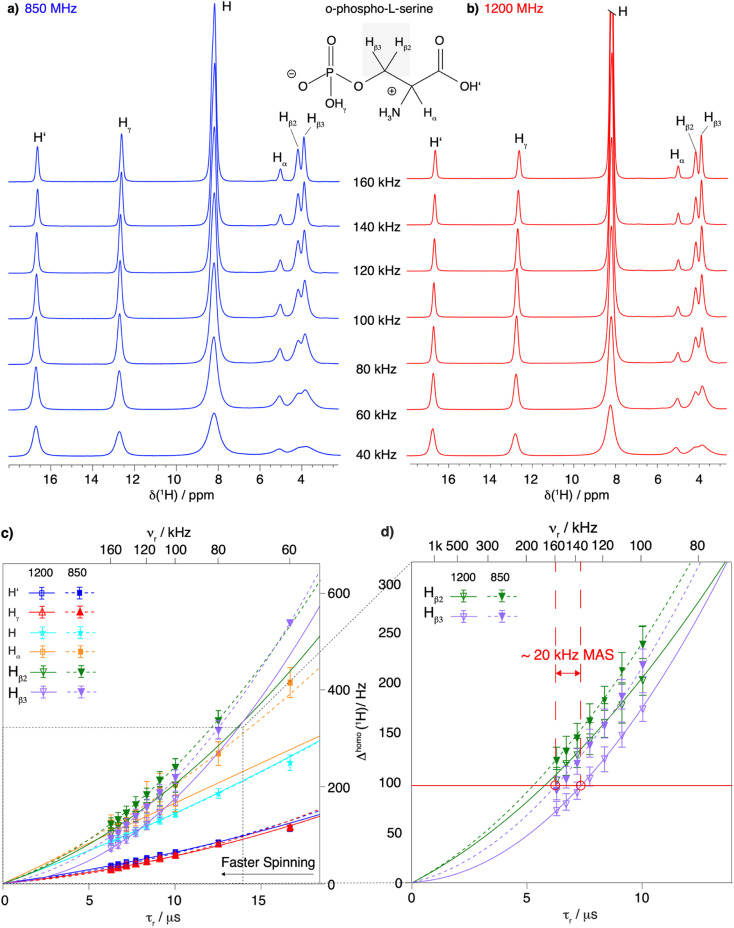
Proton line width as a function of MAS frequency for *O*-phospho-l-serine. Comparison between 1D ^1^H spectra recorded at MAS frequencies from 40 to 160 kHz and at (a) 850 MHz proton frequency and (b) 1200 MHz proton frequency. The proton resonances are labeled according to the chemical structure of *O*-phospho-l-serine in the figure. Note that the 1D spectra were not fully relaxed, explaining the intensity difference. (c) Resonance-specific homogeneous line width (*Δ*^homo^(^1^H)) as a function of the rotor period *τ*_r_ = 1/*ν*_r_ and corresponding quadratic fits of the experimental data measured at 850 MHz (dashed lines) and 1200 MHz (solid lines) proton Larmor frequency at MAS frequencies of 60 to 160 and 100 to 160 kHz MAS frequency respectively (fits shown in Fig S3 and S4†). The MAS frequency is shown on the upper axis. Error bars are calculated as described in the Materials and methods section and given as 2*σ*. At 60 kHz MAS frequency and 850 MHz proton frequency, the two protons of the CH_2_ group are not resolved enough to be measured individually. A zoom on the proton resonances of the CH_2_ group (H_β2_ and H_β3_) is shown in (d) to illustrate the similar line width obtained by spinning 20 kHz faster or going to a higher magnetic field.

We first investigated a small model system, the amino acid *O*-phospho-l-serine^47–50^ (for the chemical structure see Fig. 1) The spinning frequency dependence of its proton line widths between 40 and 160 kHz MAS at 850 and 1200 MHz proton Larmor frequency is shown in Fig. 1. Over the entire range of spinning frequencies and for both magnetic-field strengths, we observe a clear decrease in line width with increasing spinning frequency, most prominently for the CH_2_ resonances. This is also shown in Tables S5a and b† summarizing the total proton line widths (the sum of homogeneous and inhomogeneous contributions) measured at 850 and 1200 MHz proton frequency respectively. Improvements at higher magentic field by the expected factor of 1.4 (representing the ratio of the two magnetic fields) is observed for the H′ and H_γ_ spins (nomenclature in Fig. 1a), while for the remaining spins, fits of the line shapes by Lorentzian/Gaussian lines are poor as the line shapes are complex due to the higher-order terms in the AHT expansion (Fig. S8†).

To evaluate the homogeneous contribution to the total line width, 
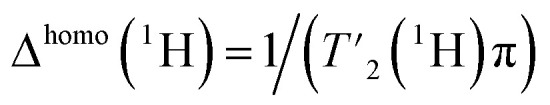
 was extracted from the Hahn-echo 1D ^1^H spectra. Fig. 1c shows the *Δ*^homo^(^1^H) dependence with *τ*_r_ = 1/*ν*_r_ and the homogeneous line widths at 850 and 1200 MHz are given respectively in Tables 1 and 2. For both magnetic fields, the proton line widths always benefit from a faster MAS frequency. The homogenous line width improvement when going from 100 to 160 kHz spinning frequency is shown to be greatest for the CH_2_ group protons (H_β2_ and H_β3_, more than a factor of two). Indeed, at MAS frequencies of 160 kHz, their homogeneous line width becomes comparable to that of the H and H_α_ protons (see Table 2 at 1200 MHz). Fig. 1c shows that, for all protons, the homogeneous line width, *Δ*^homo^(^1^H), is always lower at 1200 MHz (solid lines) than at 850 MHz (dotted lines) (see also Table 1). This effect is more pronounced for the protons of the CH_2_ group.

**Table 1:** Homogeneous proton line width *Δ*^homo^(^1^H) for *O*-phospho-l-serine measured at increasing MAS frequencies from 60 to 160 kHz and 850 MHz proton frequency. At 60 kHz MAS frequency, the two protons of the CH_2_ group are not resolved enough to be measured individually. Experimental errors (*σ*) are calculated as described in the Material and methods section

MAS frequ./kHz	*Δ* ^homo^(H′)/Hz	*Δ* ^homo^(H_γ_)/Hz	*Δ* ^homo^(H)/Hz	*Δ* ^homo^(H_α_)/Hz	*Δ* ^homo^(H_β2_)/Hz	*Δ* ^homo^(H_β3_)/Hz
160	32 ± 2	26 ± 2	84 ± 4	125 ± 8	124 ± 6	94 ± 6
150	36 ± 2	30 ± 2	92 ± 3	119 ± 7	133 ± 7	105 ± 6
140	39 ± 2	34 ± 2	98 ± 4	131 ± 7	146 ± 7	121 ± 7
130	44 ± 2	38 ± 2	107 ± 4	146 ± 8	163 ± 7	139 ± 7
120	49 ± 2	44 ± 2	118 ± 4	160 ± 30	184 ± 8	159 ± 7
110	58 ± 3	52 ± 2	130 ± 4	194 ± 18	214 ± 9	188 ± 8
100	62 ± 3	57 ± 3	144 ± 5	203 ± 10	241 ± 9	220 ± 8
80	84 ± 3	80 ± 3	185 ± 5	267 ± 12	337 ± 11	317 ± 11
60	114 ± 4	115 ± 3	248 ± 7	415 ± 16	—	—

**Table 2:** Homogeneous proton line width *Δ*^homo^(^1^H) for *O*-phospho-l-serine measured at increasing MAS frequencies from 100 to 160 kHz and 1200 MHz proton frequency. Experimental errors (*σ*) are calculated as described in the Material and methods section

MAS frequ./kHz	*Δ* ^homo^ (H′)/Hz	*Δ* ^homo^ (H_γ_)/Hz	*Δ* ^homo^ (H)/Hz	*Δ* ^homo^ (H_α_)/Hz	*Δ* ^homo^ (H_β2_)/Hz	*Δ* ^homo^ (H_β3_)/Hz
160	36 ± 2	30 ± 2	86 ± 4	105 ± 5	104 ± 7	74 ± 4
150	39 ± 2	33 ± 2	93 ± 3	112 ± 5	120 ± 7	80 ± 5
140	43 ± 3	36 ± 3	100 ± 3	115 ± 5	129 ± 8	91 ± 5
130	47 ± 2	40 ± 3	108 ± 4	130 ± 6	144 ± 9	106 ± 6
120	52 ± 2	44 ± 3	118 ± 4	136 ± 6	162 ± 9	123 ± 7
110	57 ± 3	49 ± 3	130 ± 4	155 ± 7	179 ± 10	148 ± 7
100	64 ± 3	57 ± 3	145 ± 4	164 ± 9	204 ± 11	175 ± 9

Finally, a comparison of the MAS-frequency dependence shows that it is possible to obtain a similar proton homogeneous line width at 850 MHz as at 1200 MHz, by spinning about 20 kHz faster. This is illustrated in Fig. 1d, which shows that for the proton resonances of the CH_2_ group, the homogeneous line widths at 160 kHz and 850 MHz for H_β2_ and H_β3_ are 124 ± 6 Hz and 94 ± 6 Hz, respectively, close to what would be obtained at 140 kHz and 1200 MHz, 129 ± 8 Hz and 91 ± 5 kHz, respectively (Tables 1 and 2).

Taken together, the proton line widths benefit from both a faster MAS frequency and a higher magnetic field. In addition, the experimental CH_2_ proton homogeneous line width remains to be described over the extended range up to 160 kHz MAS by a sum of a linear and a quadratic function of 1/*ν*_r_. A further linewidth improvement is expected for MAS frequencies above 160 kHz as, indeed, for the two CH_2_ resonances at 1200 MHz, the total line width (118 ± 7 and 128 ± 12 Hz, Table S5†) is still dominated by the homogeneous contribution (74 ± 7 and 104 ± 7 Hz, Table 2).

### The gain in line width in a protein sample

**Fig. 2:**
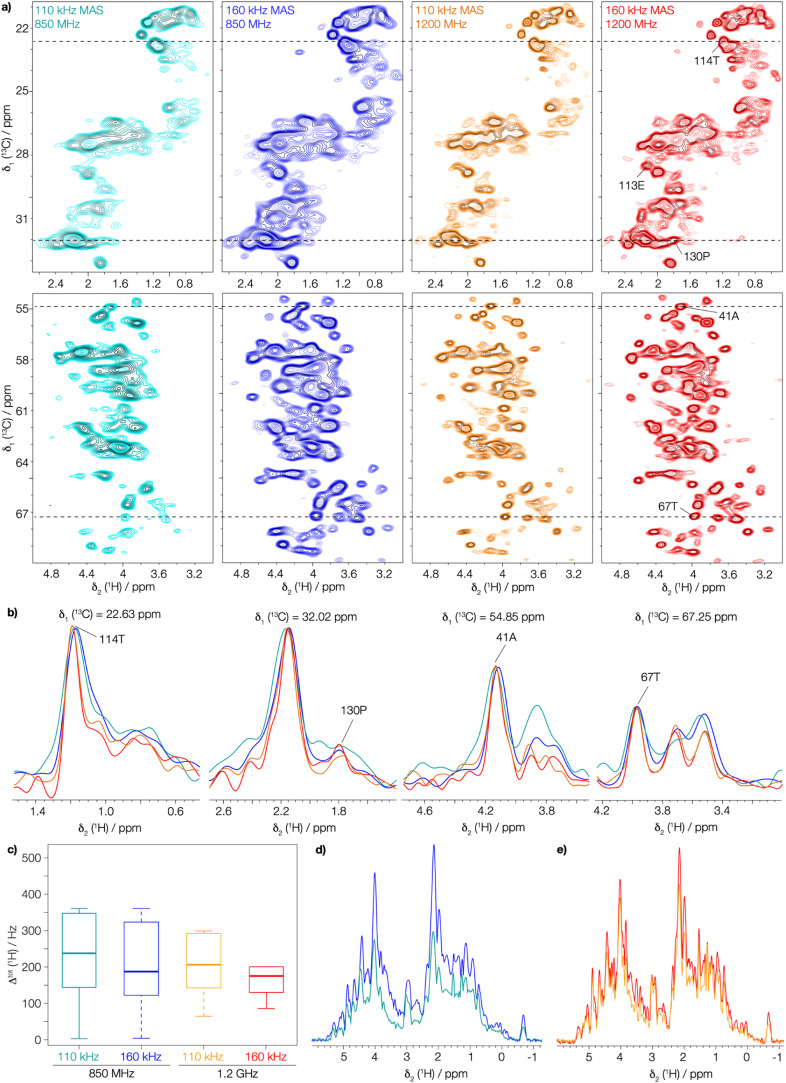
The gain in resolution at faster spinning and higher field in UL-Cp149 spectra. (a) Aliphatic regions of 2D-hCH UL-Cp149 spectra recorded at 160 kHz (red) and 110 kHz (orange) MAS frequency and 1200 MHz proton frequency and 160 kHz (blue) and 110 kHz (cyan) MAS frequency and 850 MHz proton frequency. (b) Corresponding 1D traces at the positions indicated in the 2D spectra with dashed lines. (c) Corresponding boxplot statistics of the site-specific line width of 87 peaks from the spectra in (a). The 87 peaks (Fig. S9†) were selected for evaluation are resolved in all four spectra. The thick lines in each box represent the median total proton line width (*Δ*^tot^(^1^H)), the box areas range from the 25^th^ to the 75^th^ percentile and the error bars cover the 95% confidence interval. (d) 1D-^1^H projections of the 2D-hCH spectra recorded at a magnetic field of 850 MHz and (e) 1D-^1^H projections of the 2D-hCH spectra recorded at 1200 MHz proton frequency.

Next, we investigated the assembly domain of the HBV capsid core protein (Cp149),^51,52^ for which amide-proton,^3^ as well as ^13^C and ^15^N assignments^34^ have been previously reported, but side-chain assignments are still missing. In order to compare line widths, we recorded 2D-hCH CP-based spectra of protonated and uniformly [^13^C,^15^N] labeled (UL) Cp149 at MAS frequencies of 110 and 160 kHz, at 850 MHz and 1200 MHz proton frequency. The obtained spectra are shown in Fig. 2, where the aliphatic backbone and side-chain proton–carbon correlations are displayed.

Already the inspection of the spectra in Fig. 2a allows one to appreciate the gain in resolution obtained by faster spinning and higher field, which is also clearly visible in the corresponding extracted 1D traces in Fig. 2b. To quantify this gain, we extracted the total proton line width from a selection of peaks picked in the 2D-hCH CP-based spectra. We selected 87 peaks that are resolved in all four spectra (peaks shown in Fig. S9†). The MAS frequency dependence of the line width is illustrated by the median total proton line width (*Δ*^tot^(^1^H)) which improves at 1200 MHz from 207 ± 9 Hz at 110 kHz to 176 ± 7 Hz at 160 kHz MAS frequency (thick lines in Fig. 2c). At 850 MHz, *Δ*^tot^(^1^H) improves from 237 ± 14 Hz at 110 kHz to 186 ± 8 Hz at 160 kHz MAS frequency. Thus, between 110 and 160 kHz, we observe an improvement in the total proton line width of 18% at 1200 MHz and 27% at 850 MHz. In addition, the gain in sensitivity can be seen by comparing the 1D ^1^H projections of the 2D-hCH spectra (Fig. 2d and e). This is due to the reduction in line width at faster spinning, but is also offset by the reduction in CP transfer performance at higher MAS frequency.^53^ At 1200 MHz, a gain in SNR of a factor of 1.16 is observed by spinning at 160 kHz compared to 110 kHz, while at 850 MHz the gain is 1.5. Moving to a higher magnetic field at constant spinning frequency (160 kHz) also improves the spectral resolution, as shown by comparing *Δ*^tot^((H)^1^H), from 186 ± 8 Hz at 850 MHz to 176 ± 7 Hz at 1200 MHz. The combination of faster spinning and higher magnetic field made it possible to achieve a significant reduction in the total proton line width, by a total of 35%. This is likely to enable for proton side-chain resonance assignments in many large proteins, and highlights the added benefit of combining faster MAS with higher magnetic fields.

### Relaxation times increase with faster spinning

We have seen in *O*-phospho-l-serine that the homogeneous line width decreases with increasing magnetic field and MAS frequency. Therefore, the proton transverse relaxation time 
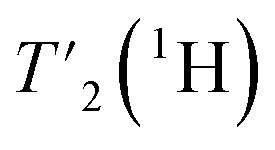
, which is a measure of the homogenous line width, should increase. This is indeed the case for the bulk 
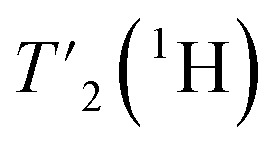
 of UL-Cp149, as shown in Fig. S7 and Table S6.† We measured in addition bulk rotating-frame proton relaxation times *T*_1ρ_((H)^1^H), as well as longitudinal relaxation *T*_1_(^1^H). Both relaxation times lengthen with increasing MAS frequency and magnetic field, as expected, for amide and aliphatic protons (Fig. S7 and Table S6†).

To go further, we measured site-specific transverse relaxation times of the aliphatic protons of UL-Cp149 using 2D-hCH 
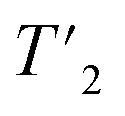
 relaxation experiments at MAS frequencies of 110 and 160 kHz at 1200 MHz proton frequency. We recorded eight 2D-hCH 
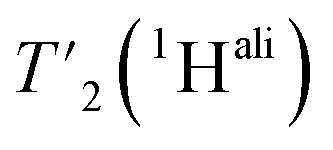
 spectra at different echo times, with the spectra recorded at 0.001 ms shown in Fig. S5 and S6† for 160 kHz and 110 kHz, respectively. The 51 residues shown in blue in Fig. S9† were selected because they are resolved and assigned in both spectra. For each of the peaks, the relaxation decay was fitted with a mono-exponential function (shown in Fig. S5 and S6†). The site-specific 
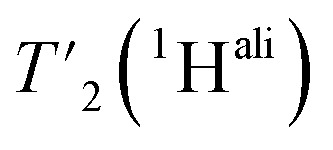
 are listed in Table S7† and the median relaxation times summarized in Table 3. Comparing the median 
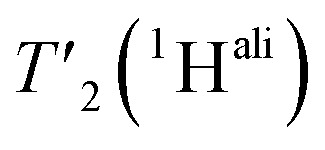
 between 110 and 160 kHz reveals an increase in relaxation times of about 50% when going to faster spinning. The longest 
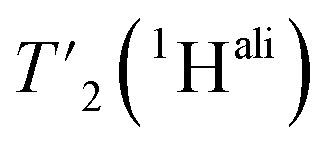
 are found for methyl protons (C**H**_3_) and **H**_α_ protons.

**Table 3:** Median 
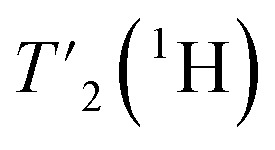
 relaxation times and *Δ*^homo^(^1^H) of UL-Cp149 aliphatic protons recorded at 1200 MHz proton frequency and spinning frequencies of 110 and 160 kHz. The spectral regions are defined by type of protons, with the C**H**_3_ spectral region between *δ*(^13^C) = 16.8–26.0 ppm, the C**H**_2_ spectral region between *δ*(^13^C) = 26.0–51.4 ppm and the H_α_ spectral region between *δ*(^13^C) = 51.4–70.0 ppm. Experimental errors (*σ*) are calculated as described in the Materials and methods section

	110 kHz MAS		160 kHz MAS	
	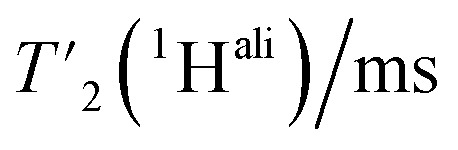	*Δ* ^homo^(^1^H^ali^)/Hz	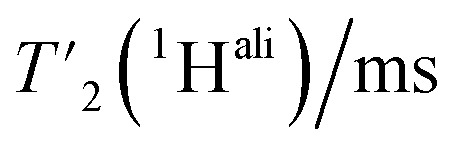	*Δ* ^homo^(^1^H^ali^)/Hz
**H**_all_	4.3 ± 1.1	77 ± 16	6.6 ± 1.0	50 ± 7
**H**_α_	4.5 ± 1.1	71 ± 17	6.3 ± 1.1	50 ± 7
C**H**_2_	3.4 ± 0.6	94 ± 16	5.3 ± 0.7	60 ± 7
C**H**_3_	4.9 ± 1.1	64 ± 13	8.1 ± 1.7	39 ± 6

**Fig. 3:**
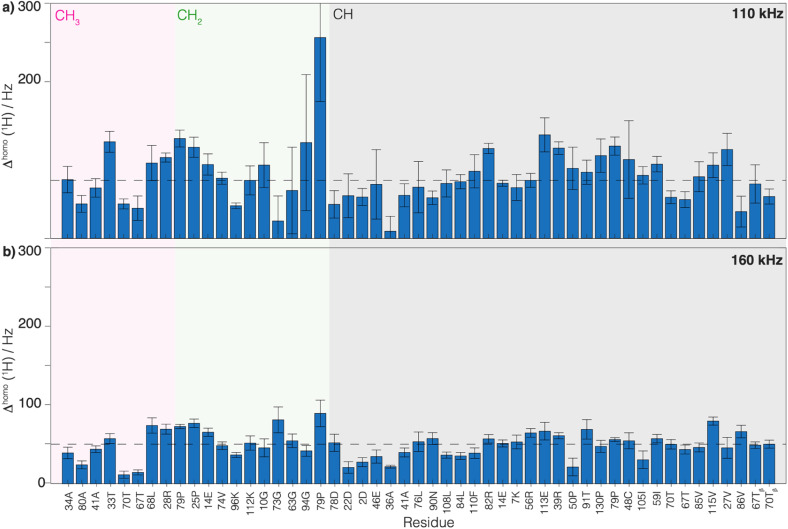
Comparison of the homogeneous proton line widths measured at 1200 MHz proton frequency at 160 and 110 kHz MAS. Site-specific homogeneous proton line width of UL-Cp149 extracted from 2D-hCH CP-based spectra measured at (a) 110 kHz and (b) 160 kHz MAS frequency and at 1200 MHz proton frequency. In both plots, the dotted line represents the median homogeneous proton line width. The spectral regions are defined as follows: the C**H**_3_ spectral region between *δ*(^13^C) = 16.8–26.0 ppm (grey area), the C**H**_2_ spectral region between *δ*(^13^C) = 26.0–51.4 ppm (green area) and the C**H** spectral region between *δ*(^13^C) = 51.4–70.0 ppm (pink area). The error bars are calculated according to the description in the Material and methods section and given as 2*σ*. The residues labeled with a star in the CH region are C**H**_2_ groups.

We then extracted the site-specific homogeneous line width of the aliphatic protons, 
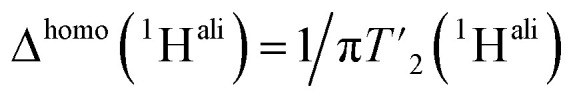
, from the measured 
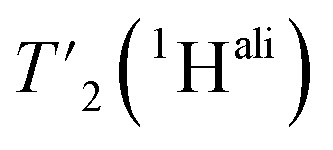
 relaxation times of the 51 residues, at 160 and 110 kHz MAS frequency. The site-specific homogeneous proton line widths are shown in Fig. 3a and b respectively and listed in Table S7.† As for the model compound, the median homogeneous line width decreases at higher MAS frequencies, from 77 ± 16 at 110 to 50 ± 7 Hz at 160 kHz MAS frequency, corresponding to a reduction of a factor 1.5. This is close to the expected improvement which corresponds to the inverse ratio of the spinning frequencies, 160 kHz/110 kHz = 1.4.

Thus, the reduction of the homogeneous line width is higher than the reduction of the total line width (reduction by a factor of about 1.2, see above). This is due to the larger inhomogeneous than homogeneous contribution to the total line width, which is independent of the spinning frequency. Thus, we conclude that the dominant contribution to the total line width at 160 kHz comes from the inhomogeneous contributions (median *Δ*^inhomo^ = *Δ*^tot^ − *Δ*^homo^ = 126 Hz, for the selected 51 residues). It accounts in UL-Cp149 for two-thirds of the total line width and limits the amount of improvement that can be achieved by spinning even faster. One should note that Cp149 has four different molecules in the asymmetric unit, resulting in some resonances being split into four resolved peaks^3^ and, for other resonances, unresolved peak quadrupling might contribute to, or even dominate, the inhomogeneous line width in this sample. Note that the homogeneous line width is larger for the CH_2_ spins than for the H_α_ or CH_3_ (Table 3) due to the stronger dipolar coupling they experience. For these spin systems, the advantage of going to a higher field and spinning faster is more pronounced.

### Aliphatic proton assignments at 1200 MHz and 160 kHz MAS

Using fastest spinning and highest magnetic field, we recorded backbone and side-chain spectra allowing to assign the aliphatic protons. We first used a 3D-hNCAHA^19^ spectrum of UL-Cp149 recorded at 160 kHz MAS and 1200 MHz proton frequency (shown in Fig. S10†) to assign the H_α_ resonances based on the ^15^N and ^13^C assignments (Lecoq *et al.*^34^) 51% of the H_α_ protons with a ^13^C assignment from Lecoq *et al.*^34^ could be assigned from this spectrum. The assigned residues are labeled in Fig. S10,† where the right panel shows a single 3D plane extracted at *δ*_1_(^15^N) = 114.67 ppm as an example. To further assign the aliphatic side-chain protons, we recorded a 3D-hCCH total through bond correlation spectroscopy (TOBSY) spectrum.^10,19,20,38,54,55^ We used as mixing sequence a C9_57_ mixing block^38^ to maximize the ^13^C–^13^C polarization transfer efficiency (pulse sequence shown in Fig. S2†). A mixing time of 15 ms, slightly longer than 1/2*J*_CC_ ∼ 11 ms, was used as a compromise to obtain both one- and two-bond carbon *J*-coupling transfers. A 10 ms z-filter allowed to remove unwanted coherences from the spectrum without significant magnetization loss. Note that, 3D-hCCH and -HcCH TOBSY experiments were also recorded using a WALTZ-16 block during the TOBSY transfer with an irradiation amplitude of 30 kHz (giving the strongest signals in the 1D and 2D versions of the 3D-hCCH TOBSY used for optimization, see Fig. S11 and S12†). The 3D-hCCH TOBSY-C9 was further used for side-chain proton assignments, since it resulted in more cross peaks than the WALTZ-16 versions.

**Fig. 4:**
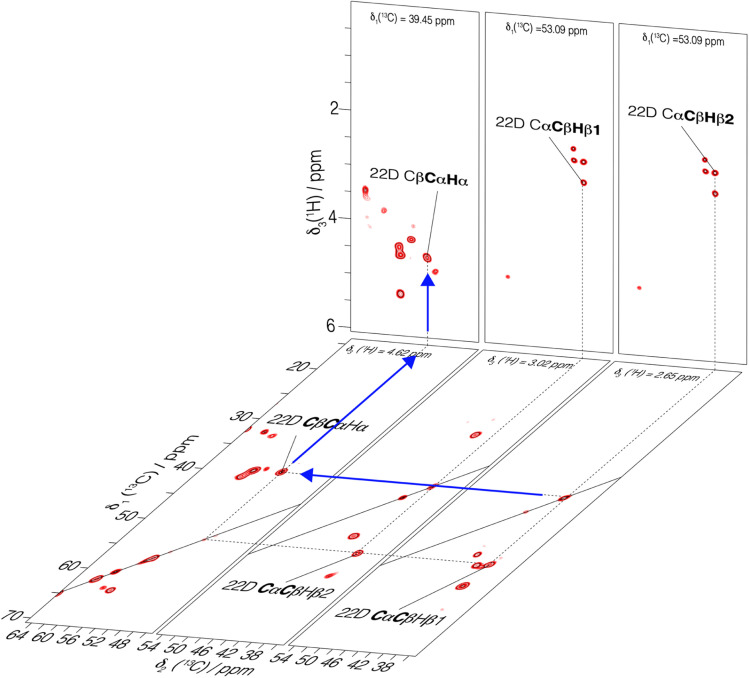
3D-hCCH TOBSY-C9 spectrum of UL-Cp149 recorded at 160 kHz MAS frequency and 1200 MHz proton frequency. Selected 2D-hCCh and hcCH planes are shown. An example of assignment of H_α_ of residue 22D is represented with blue arrows where the known carbon chemical shifts^34^ served as basis to assign the bound protons.

Fig. 4 shows extracted planes of the 3D-hCCH TOBSY-C9 spectrum of UL-Cp149 recorded at 1200 MHz proton frequency and 160 kHz MAS frequency. The assignment strategy for assigning the aliphatic protons using the 3D-hCCH spectrum is shown for residue 22D. Based on the known ^13^C assignments from Lecoq *et al.*^34^ the resonance in the CC plane resulting from one-bond TOBSY magnetization transfer could be assigned, identifying the bound aliphatic proton. Following this strategy, the H_α_ (4.62 ppm), H_β1_ (3.02 ppm) and H_β2_ (2.65 ppm) resonances of 22D could be assigned thanks to their corresponding carbon assignment (Fig. 4).

**Fig. 5:**
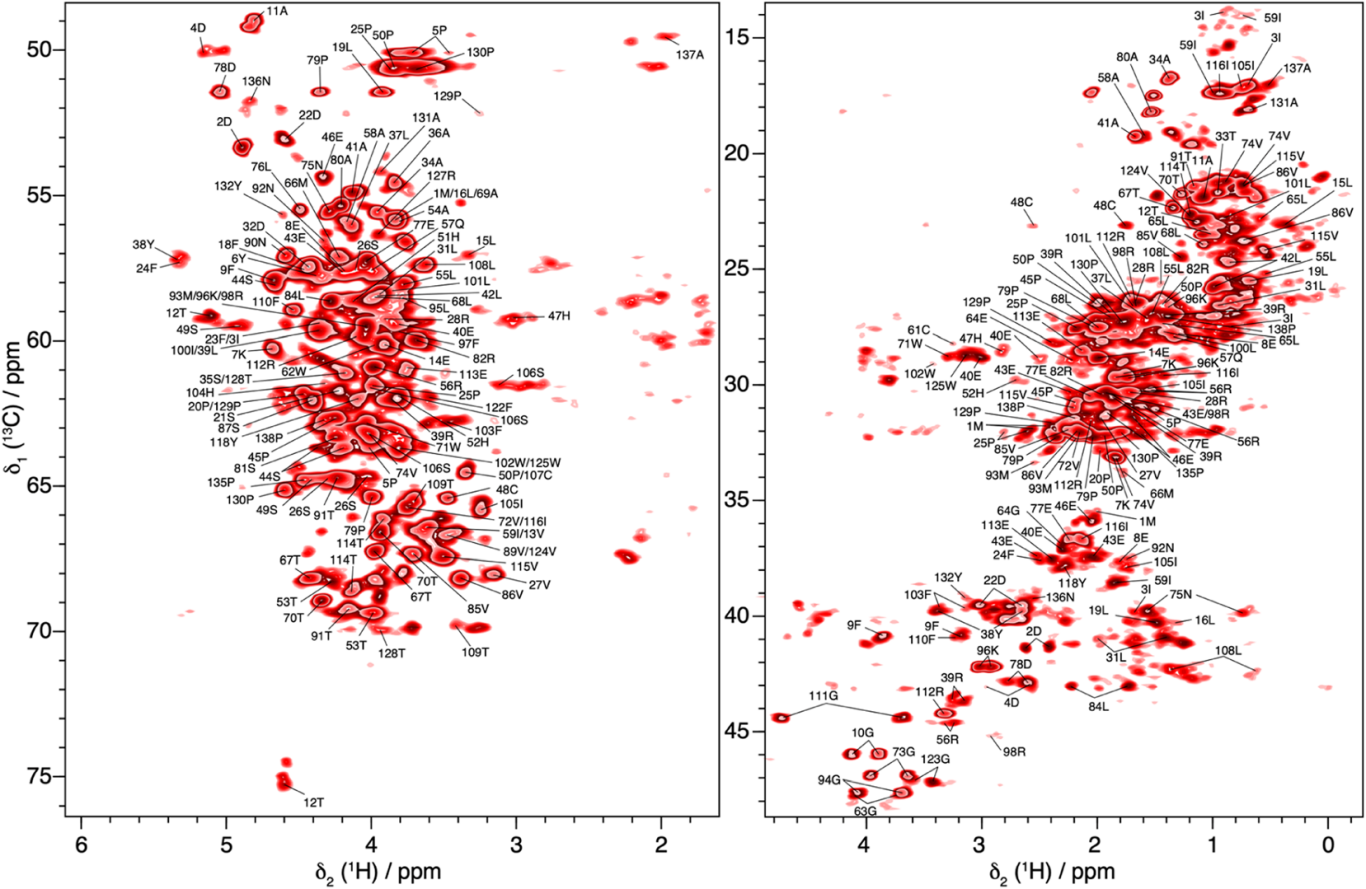
2D-hCH spectrum of UL-Cp149 measured at 160 kHz MAS frequency and 1200 MHz proton frequency, with aliphatic proton assignments obtained from the 3D-hNCH and hCCH experiments.

In total, 61% of the aliphatic protons could be assigned, including 81% of H_α_ and 52% of the CH_2_ and CH_3_ groups (see Fig. S13†). Note that the protons marked with an asterisk in Fig. S13† could not be assigned, because their directly bonded carbon had not been assigned previously. They are however taken into account in calculating the percentages. Furthermore, the fastest spinning allows to resolve the two diastereotopic proton resonances in about half of the assigned CH_2_ groups. This is shown as an example in Fig. 4, where the two beta proton resonances of 22D (H_β1_ and H_β2_) are resolved in the hcCH plane of the 3D-hCCH TOBSY spectrum. The total assignment is shown in the 2D-hCH spectrum of UL-Cp149 in Fig. 5 below.

## Discussion and conclusion

We describe the benefits of fast magic-angle spinning at 160 kHz, combined with high magnetic field, to proton-detected solid-state NMR. The basic aspects are described semi-quantitatively for a model system, and the findings are confirmed for the hepatitis B viral capsid protein, in which we achieve a large part of the side-chain assignments using proton-detected MAS solid-state NMR spectroscopy at 160 kHz MAS and 1200 MHz. We quantify the gain in resolution obtained at this spinning speed compared to the more standard 110 kHz MAS and observe an improvement in the total proton line width of 18% at 1200 MHz and 27% at 850 MHz. Subsequently, the gain in SNR is found to be 1.16 and 1.5 respectively. The reduction of the homogeneous line width and consequently of the total line width by faster spinning, together with the use of a higher static magnetic field of 1200 MHz, leads to an improvement of the total line width of 35%. Thus, a gain in resolution is achieved, notably of the side-chain protons, which suffer most from signal overlap at 110 kHz. The methylene protons benefit the most from faster spinning because they are involved in a stronger coupling regime compared to amide or H_α_ protons. Indeed, they are not only close in chemical shift to their neighbors but also often surrounded by numerous additional protons with similar chemical shift, forming a strong dipolar coupling network.

The combination of spinning at 160 kHz MAS frequency combined with high magnetic field of 1200 MHz proton Larmor frequency allowed us to assign 61% of the aliphatic protons of the HBV capsid protein and 67% of aliphatic protons having a directly bonded carbon assigned.

This information is crucial as these protons are often located at protein interfaces and their observation will further allow to characterize interactions between proteins, or to nucleic acids or small ligands. We conclude that ^1^H-detected MAS-NMR at spinning frequencies of 160 kHz and faster allows the resolution of protein side-chain resonances in fully protonated systems which carry crucial information through the contacts they make at protein–protein, protein–drug or protein–RNA (DNA) interfaces.

## Data availability

The aliphatic proton assignment of the Cp149 capsid protein has been deposited in the BioMagResBank (BMRB) under the accession number 52014. The original contributions presented in the study are included in the article/ESI,† further inquiries are available from the corresponding authors upon reasonable request.

## Author contributions

Conceptualization: BHM, MC, AB, TW, AS; AAM, BHM, AAM; formal analysis: MC, DL, AAM, TV, VR; funding acquisition: BHM, AB; investigation: MC, DL, AAM, TW, VR, LL AB, AS, BHM; methodology: BHM, MC, AB, TW, AS; project administration: BHM; resources: BHM, AB, AS; supervision: BHM, AB, AS; writing – original draft: MC, AAM, TW; writing – review & editing: all authors.

## Conflicts of interest

The authors declare no conflict of interest.

## Supplementary Material

SC-014-D3SC03539E-s001
