# Supplementary material for: High and fast: NMR protein–proton side-chain assignments at 160 kHz and 1.2 GHz
Source: Chem Sci. 2023 Sep 20;14(39):10824–34. doi: 10.1039/d3sc03539e (PMC10566471; doi:10.1039/d3sc03539e)
Supplement: SC-014-D3SC03539E-s001 [file SC-014-D3SC03539E-s001.pdf]

Electronic Supplementary Material (ESI) for Chemical Science.
This journal is © The Royal Society of Chemistry 2023

Supplementary information for

High and fast: NMR protein-proton side-chain assignments at 160 kHz and 1.2 GHz

Morgane Callon[#,1,*], Dominique Luder[1], Alexander A. Malär[#,1,a,†], Thomas Wiegand[1,b,c,†], Vaclav Rimal[1], Lauriane Lecoq[5], Anja Böckmann[5], Ago Samoson[6] and Beat H. Meier[1*]

[1] Physical Chemistry, ETH Zürich, 8093 Zürich, Switzerland

[2] Molecular Microbiology and Structural Biochemistry (MMSB) UMR 5086 CNRS/Université de Lyon, Labex Ecofect, 7 passage du Vercors, 69367 Lyon, France

[6] Institute of Cybernetics, Spin Design Laboratory, Tallinn University of Technology, Tallinn, Estonia

[a] Present address: Fraunhofer Headquarters, Hansastr. 27c, 80686 Munich, Germany

[b] Present address: Institute of Technical and Macromolecular Chemistry, RWTH Aachen University, Worringerweg 2, 52074 Aachen, Germany

[c] Present address: Max-Planck-Institute for Chemical Energy Conversion, Stiftstr. 34-36, 45470 Mülheim an der Ruhr, Germany

[#] These authors contributed equally

[*]Corresponding authors: MC (morgane.callon@phys.chem.ethz.ch) and BHM (beme@ethz.ch)

Supplementary figures

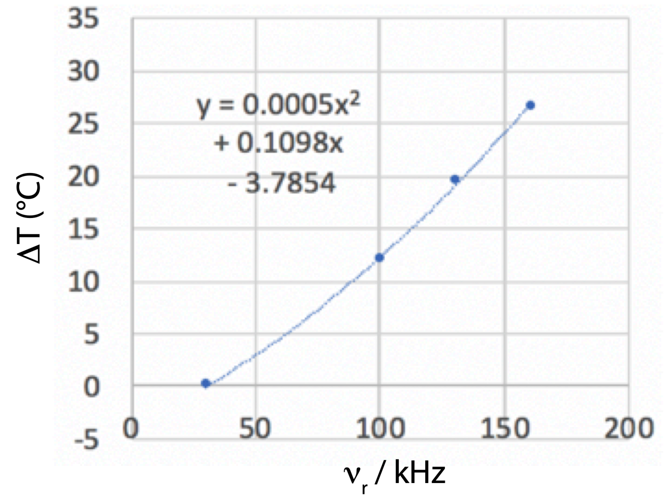

FIGURE S1 Increase in sample temperature (ΔT) as a function of the MAS frequency (ν_r), for a sample in a 0.5 mm rotor, in a range of MAS frequency from 30 to 160 kHz, measured on lead Nitride.

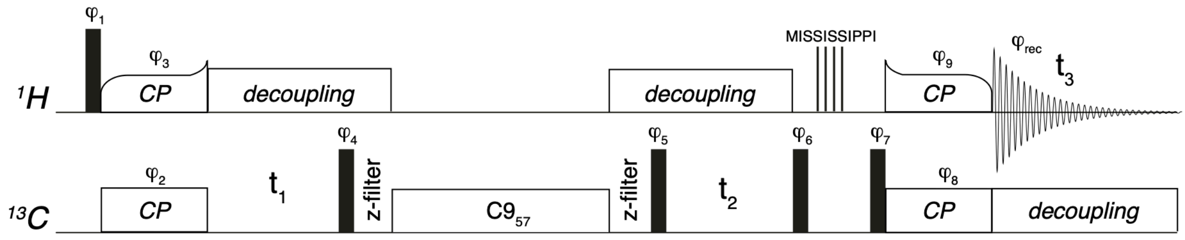

FIGURE S2 Pulse sequence scheme for the 3D-hCCH TOBSY experiment. Black narrow boxes represent π/2 pulses. The hard pulse durations were 1.667 and 2.5 μs for ^{1}H and ^{13}C respectively. Cross-polarisation (CP) transfers details are summarized in Table S4. The z-filter was set to 10 ms. Pulse phases are: $\varphi_1 = 2(y), 2(-y); \varphi_2 = x, -x; \varphi_3 = x; \varphi_4 = y; \varphi_5 = x; \varphi_6 = -x; \varphi_7 = y; \varphi_8 = x; \varphi_9 = x; \varphi_{rec} = x, -x, -x, x.$ Quadrature detection was done using States-TPPI[56] by incrementing φ_5

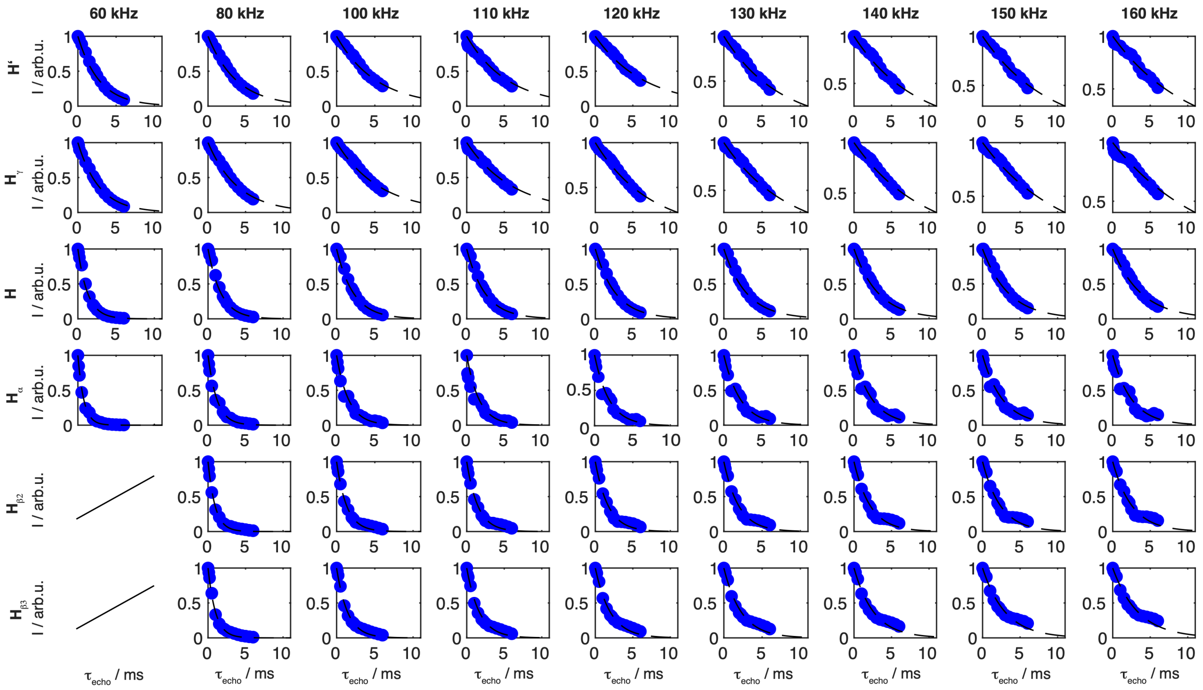

FIGURE S3 Relaxation decay traces for $T_2'(^1H)$ determination of *O*-phospho-L-serine, using Hahn-Echo experiments recorded in an 0.5 mm rotor between 60 and160 kHz MAS at 850 MHz proton frequency. The corresponding mono-exponential fits are given as black lines. At 60 kHz, the two protons of the CH_2 groups are not enough resolved to be able to measure their relaxations individually (see Fig. 2).

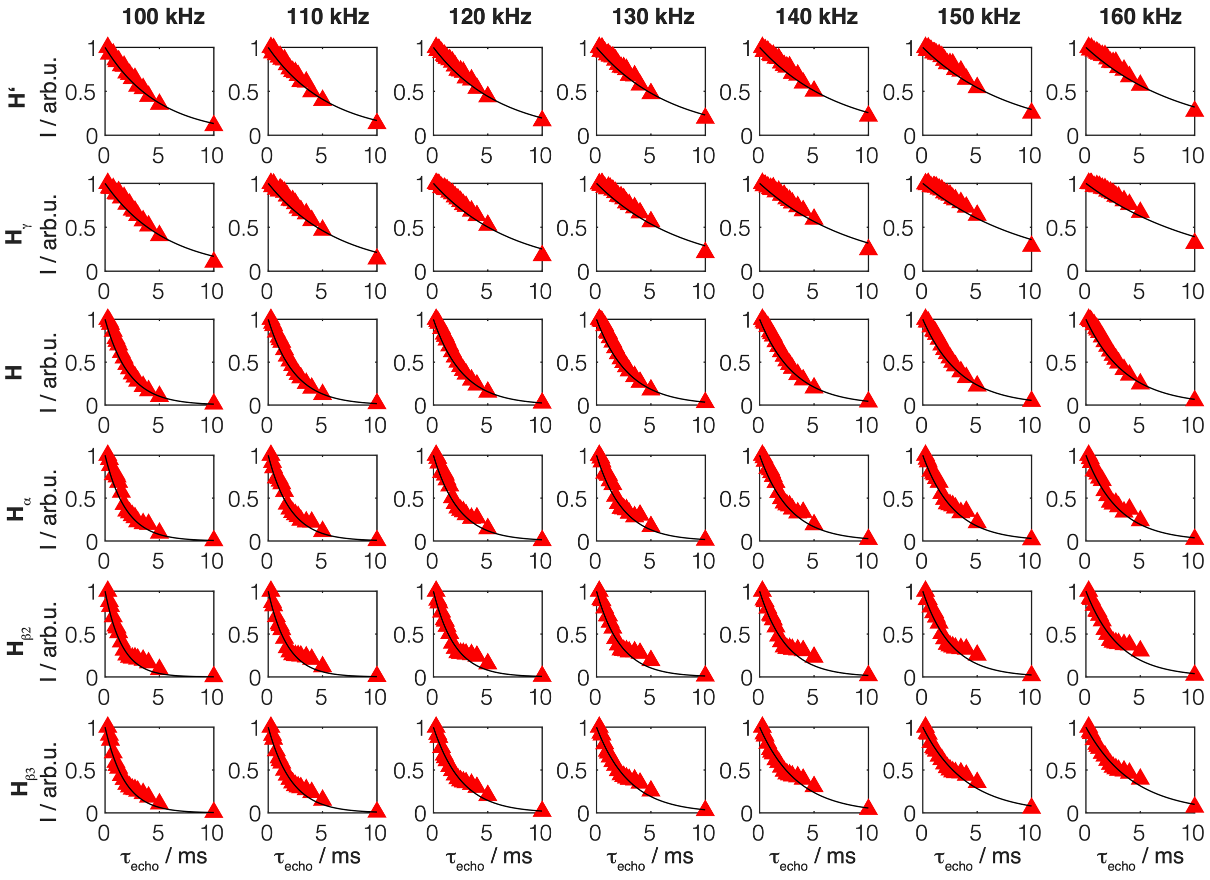

FIGURE S4 Relaxation decay traces for $T_2'(^1H)$ determination of o-o L ser, using Hahn-Echo experiments recorded in an 0.5 mm rotor between 100 and160 kHz MAS at 1200 MHz proton frequency. The corresponding mono-exponential fits are given as black lines.

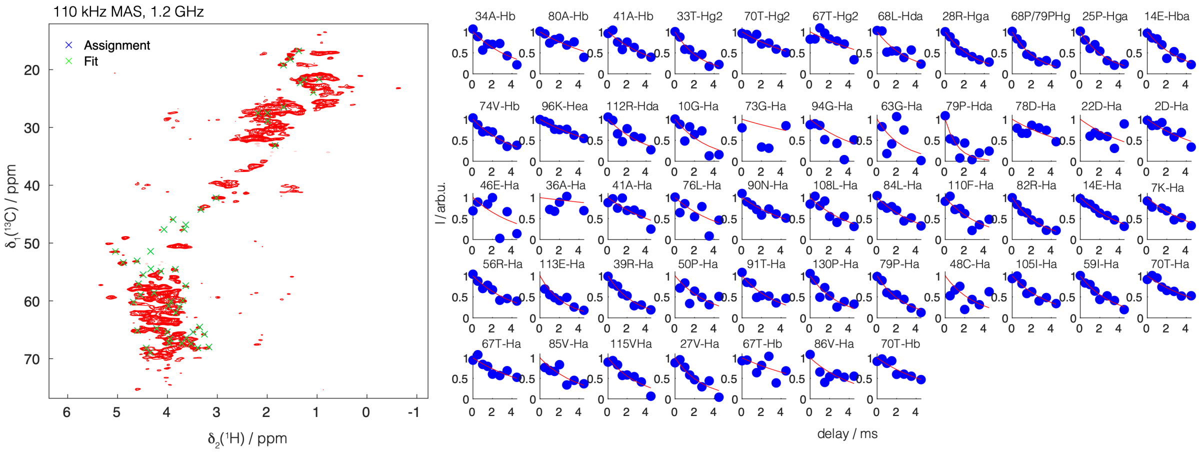

FIGURE S5 Site specific homogeneous line width measurement for UL-Cp149 at 110 kHz MAS frequency and 1200 MHz proton frequency. Left: 2D-hCH spectrum recorded with 1 µs spin echo time where the assigned peaks are shown with blue crosses and the corresponding fitted position with a green cross (overlapping, indicating a good fit). Right: corresponding extracted site specific $T_2'(^1H)$ relaxation traces with corresponding mono-exponential fits.

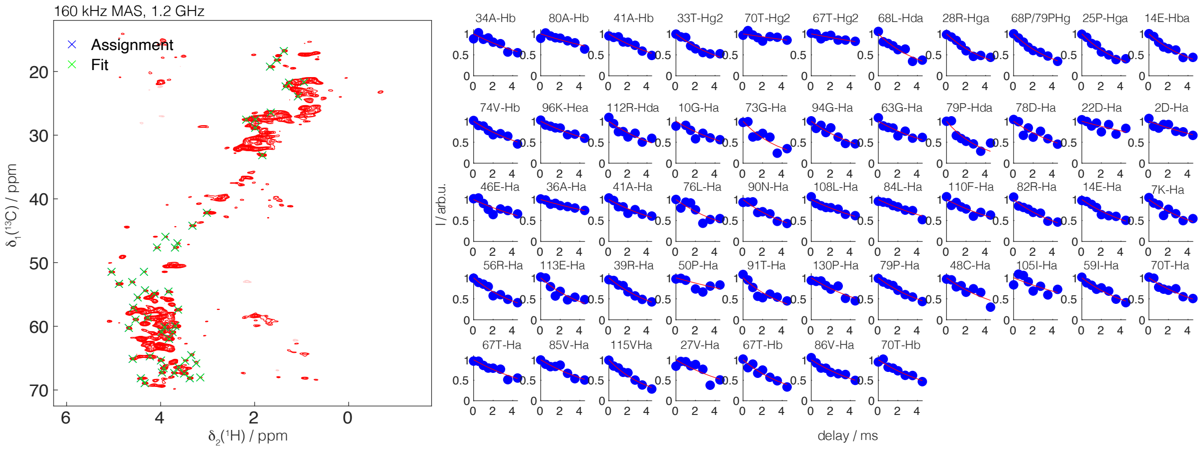

FIGURE S6 Site specific homogeneous line width for UL-Cp149 at 160 kHz MAS frequency and 1200 MHz proton frequency. Left: 2D-hCH spectrum recorded with 1 µs spin echo time where the assigned peaks are shown with blue crosses and the corresponding fitted position with a green cross (overlapping, indicating a good fit). Right: corresponding extracted site specific $T_2'(^1H)$ relaxation traces with corresponding mono-exponential fits.

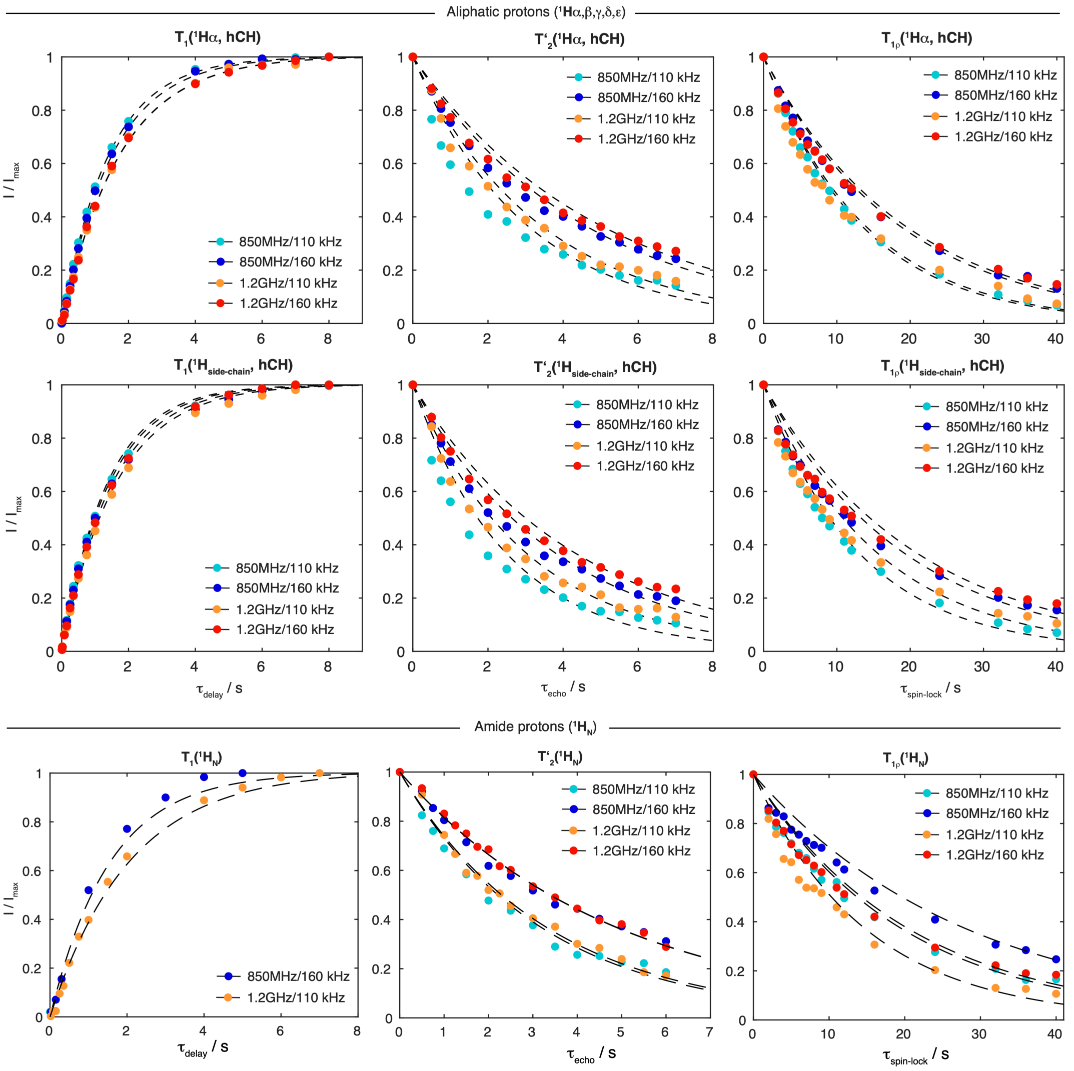

FIGURE S7 Bulk relaxation decay traces for $T_2'(^1H)$, $T_{1\rho}(^1H)$ and $T_1(^1H)$ determination for UL-Cp149, in an 0.5 mm rotor between 100 and160 kHz MAS at proton frequencies of 850 and 1200 MHz. The corresponding mono-exponential fits are given as black dashed lines.

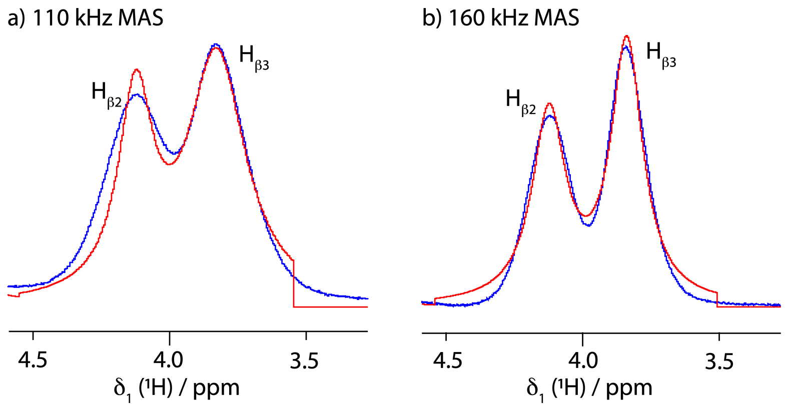

FIGURE S8 1D ^{1}H spectra (blue) of *O*-phospho-L-serine recorded at a) 110 and b) 160 kHz MAS frequency and 850 MHz proton frequency. The fits of the line shapes Lorentz/Gauss lines are shown in red.

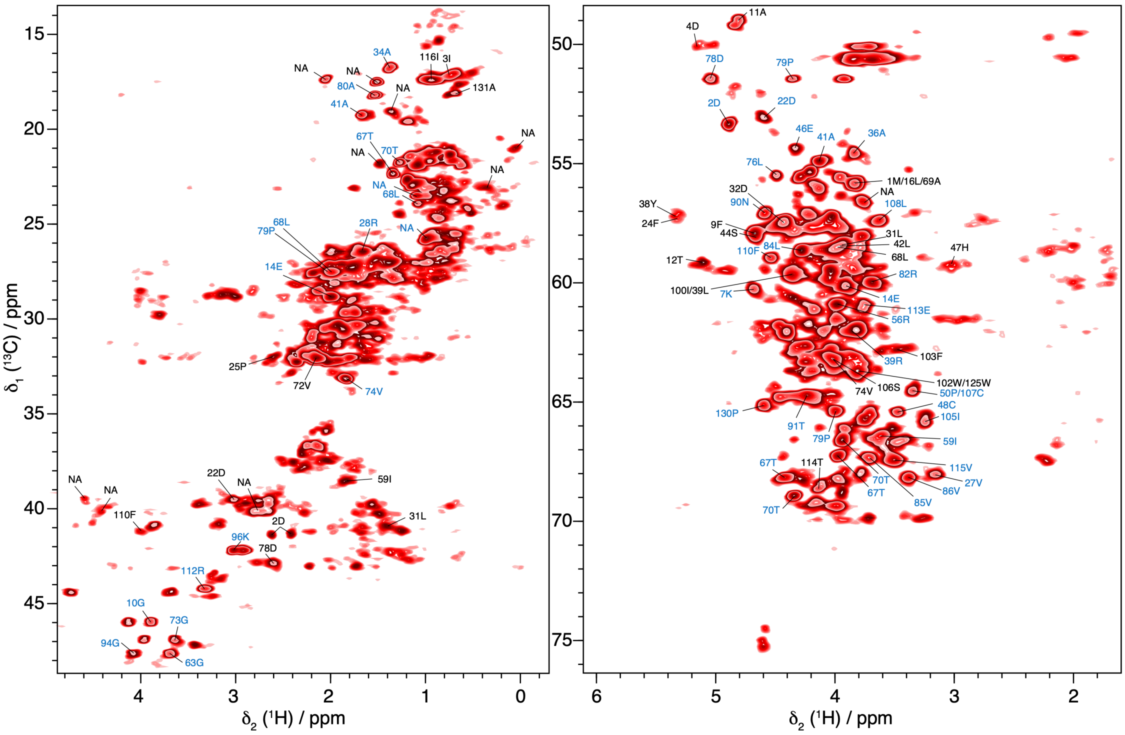

FIGURE S9 2D-hCH CP based spectrum of UL-Cp149 recorded at 160 kHz MAS frequency at 1200 MHz proton frequency. The peaks selected to measure the total (Fig. 2) proton line width are labeled. Among them, the homogeneous line width (Fig. 3) was measured on the 51 peaks labeled in blue. The peaks labeled NA are not assigned.

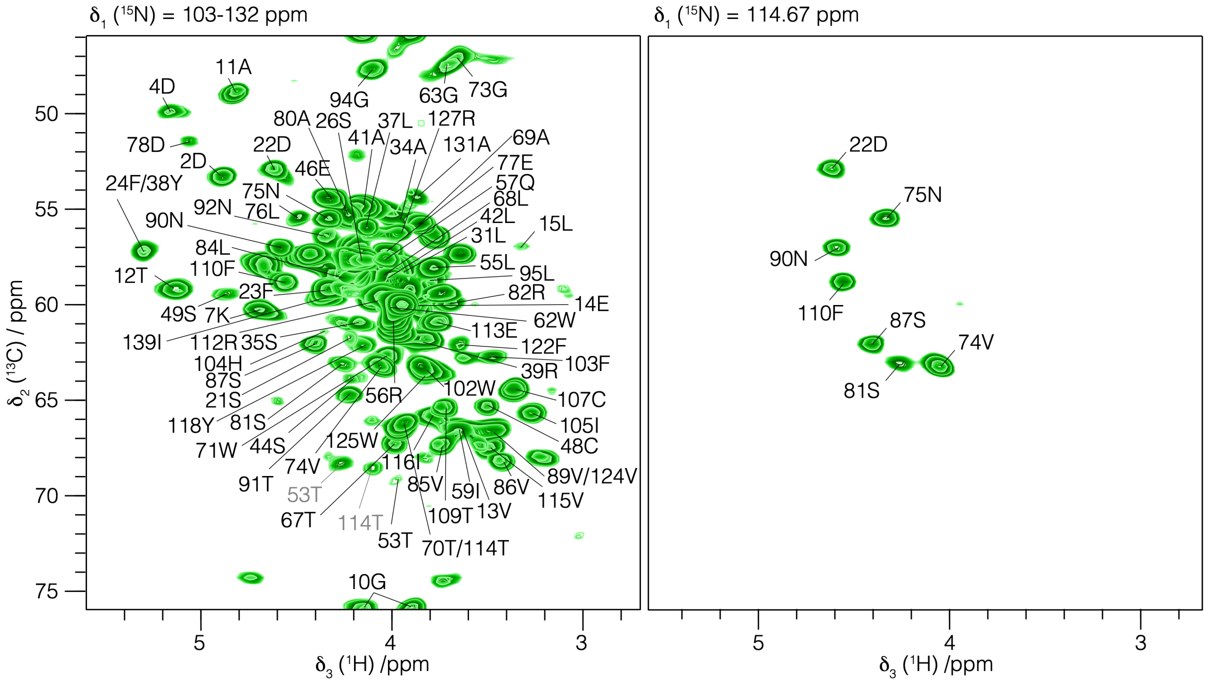

FIGURE S10 3D-hNCAHA spectrum of UL-Cp149 recorded at 160 kHz MAS frequency and 1200 MHz proton frequency. The amino-acid residues for which the H_α resonance was assigned are labeled. The residues labeled in grey correspond to the correlation between ^{15}N, C_β and H_β of threonine residues. The right shows an extracted plan at δ_1 (^{15}N) = 114.67 ppm.

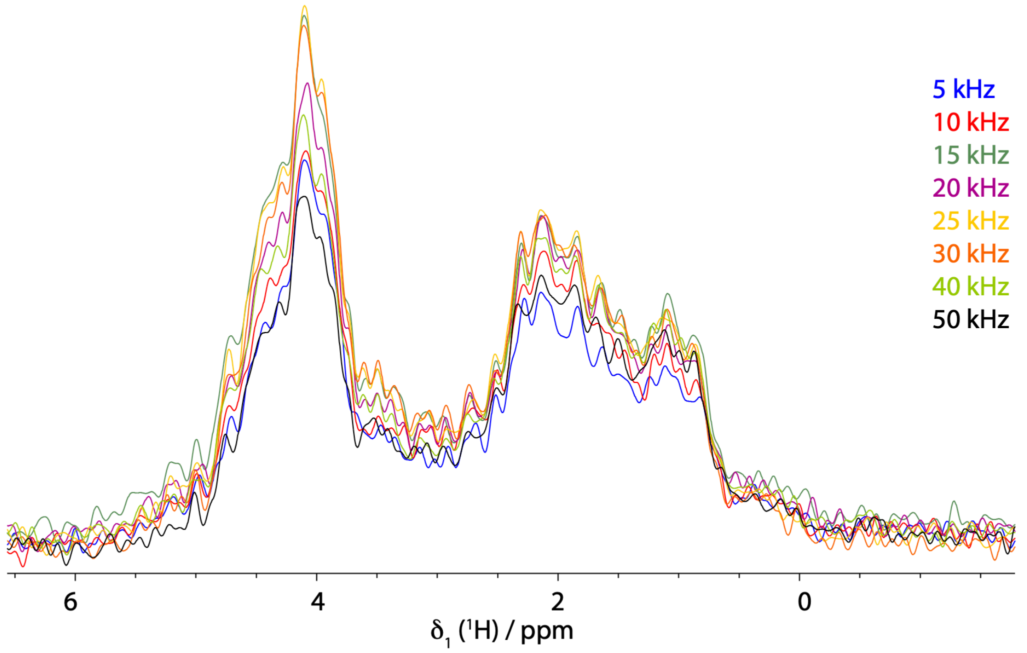

FIGURE S11 WALTZ-16 irradiation optimization for TOBSY transfer in 3D-hCCH and HcCH experiments. 1D hccH TOBSY-WALTZ16 spectra of UL-Cp149 recorded at 160 kHz MAS frequency and at proton frequency of 1200 MHz at different WALTZ-16 irradiation frequency: 5, 10, 15, 20, 25, 30, 40 and 50 kHz.

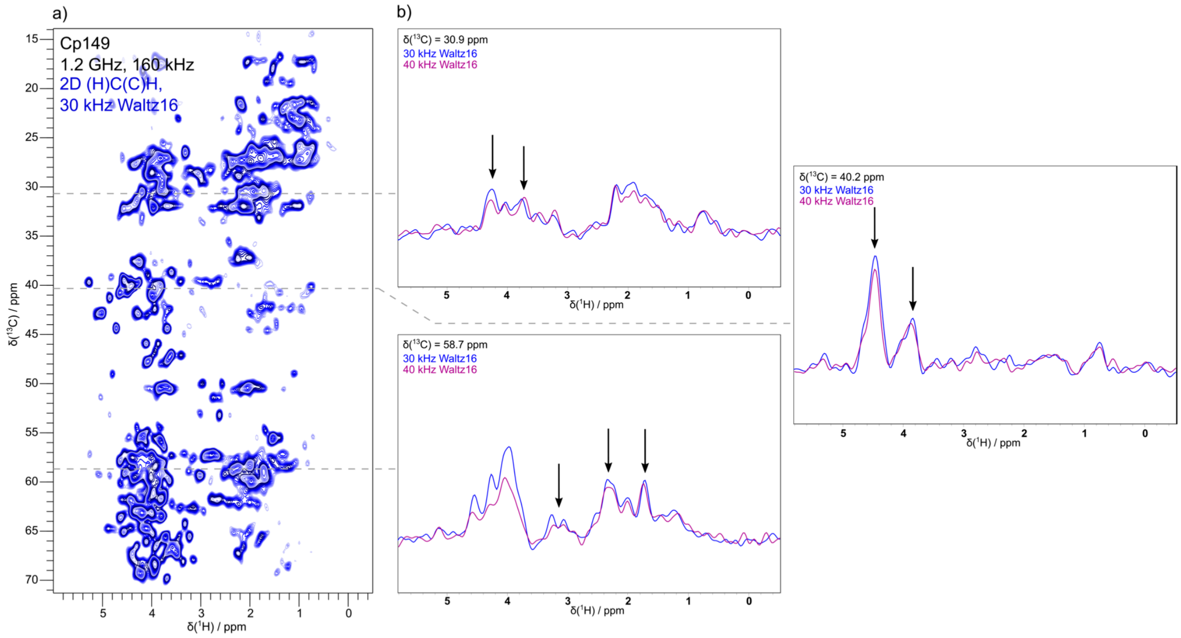

FIGURE S12 WALTZ-16 irradiation optimization for TOBSY transfer in 3D-hCCH and 3D-HcCH experiments. **a)** 2D hCcH TOBSY-WALTZ16 spectrum of UL-Cp149 recorded at 160 kHz MAS frequency and at proton frequency of 1200 MHz at a WALTZ-16 irradiation frequency of 30 kHz. **b)** 1D extracted traced of 2D-hCcH TOBSY-WALTZ16 spectra of UL-Cp149 recorded at 160 kHz MAS frequency and at proton frequency of 1200 MHz at WALTZ-16 irradiation frequencies of 30 and 40 kHz. The peaks indicated by arrows corresponds to correlation between the first carbon and the last proton of the hCCH experiment.

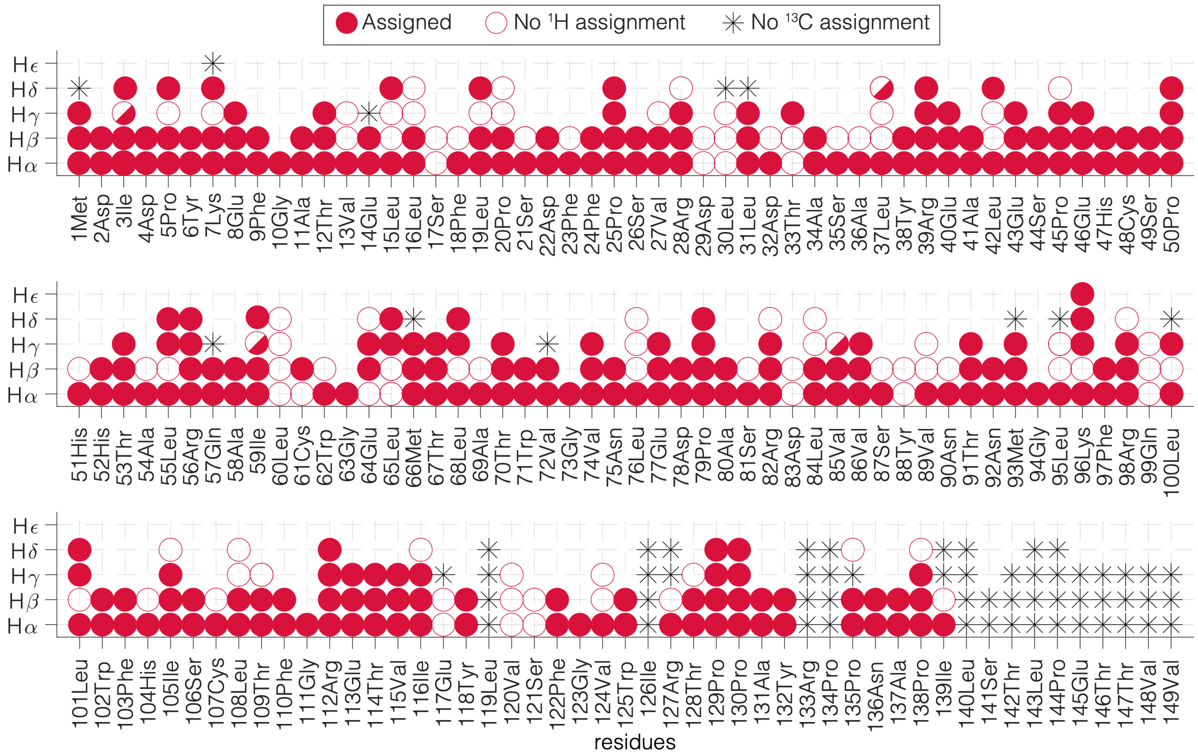

FIGURE S13 Assignment of UL-Cp149 aliphatic protons using the 3D-hNCAHA and the 3D-hCCH and HcCH TOBSY experiments experiment. 61 % of the aliphatic protons could be assigned and are colored in red, among which 81 % of Hα and 52 % of side chain protons (Hβ, Hγ, Hδ and Hε). The residues for which the carbon assignment is missing are shown with asterisks.

Supplementary Tables

TABLE S1. Experimental table for solid-state NMR acquisition and pulse program parameters used for the 2D-hCH CP-based experiments.

Experiment	2D hCH CP	2D hCH CP	2D hCH CP	2D hCH CP
^{1}H Field / MHz	850	850	1200	1200
MAS frequency / kHz	110	160	110	160
Number of scans	48	48	48	48
Experimental time	16h26	16h26	28h07	28h08
t_1 increment	640	640	904	90448
Sweep width (t_1) / ppm	70	70	70	70
Acquisition time (t_1) / ms	21.39	21.39	21.38	21.38
t_2 increment	2048	2048	4096	4096
Sweep width (t_2) / ppm	46.68	46.68	36.96	36.96
Acquisition time (t_2) / ms	25.80	25.80	36.86	36.86
^{1}H decoupling (swftppm) / kHz	10	10	10	10
^{13}C decoupling (WALTZ64) / kHz	5	5	5	5
MISSISSIPPI (120ms) / kHz	20	20	20	20
Interscan delay / s	1.76	1.86	2.1	2.1
**Transfer 1**	**HC dipolar**	**HC dipolar**	**HC dipolar**	**HC dipolar**
^{1}H field / kHz	132	131	89	125
^{13}C field / kHz	24	24	20	31
Shape	Tangent ^{1}H	Tangent ^{1}H	Tangent ^{1}H	Tangent ^{1}H
CP contact time / ms	0.8	0.8	0.8	0.8
**Transfer 2**	**CH dipolar**	**CH dipolar**	**CH dipolar**	**CH dipolar**
^{1}H field / kHz	120	124	89	125
^{13}C field / kHz	24	24	20	31
Shape	Tangent ^{1}H	Tangent ^{1}H	Tangent ^{1}H	Tangent ^{1}H
CP contact time / ms	800	800	800	800
Carrier ^{13}C / ppm	36.22	36.22	38.55	35.55
Carrier ^{1}H / ppm	1.02	1.02	2.56	0.43

TABLE S2. Experimental table for solid-state NMR acquisition and pulse program parameters used for the 2D-hCH T_2' experiments on Cp14.

Experiment	2D hCH CP T_2'	2D hCH CP T_2'
^{1}H Field / MHz	1200	1200
MAS frequency / kHz	110	160
Number of scans	16	24
Experimental time	43h21	65h45
t_1 increment	540	540
Spectral width (t_1) / ppm	70	70
Acquisition time (t_1) / ms	12.77	12.77
t_2 increment	4096	4096
Spectral width (t_2) / ppm	46.25	46.25
Acquisition time (t_2) / ms	36.86	36.86
^{1}H decoupling (swftppm) / kHz	10	10
^{13}C decoupling (WALTZ64) / kHz	5	5
MISSISSIPPI (120ms) / kHz	20	20
Interscan delay / s	2.1	2.1
**Transfer 1**	**HC dipolar**	**HC dipolar**
^{1}H field / kHz	93	128
^{13}C field / kHz	21	31
Shape	Tangent ^{1}H	Tangent ^{1}H
CP contact time / ms	0.8	0.8
**Transfer 2**	**CH dipolar**	**CH dipolar**
^{1}H field / kHz	89	128
^{13}C field / kHz	21	31
Shape	Tangent ^{1}H	Tangent ^{1}H
CP contact time / ms	0.8	0.8
Mixing time / ms	(0.001, 0.5, 1, 1.5, 2, 2.75, 3.5, 4.5)	(0.001, 0.5, 1, 1.5, 2, 2.75, 3.5, 4.5)
Carrier ^{13}C / ppm	37.44	37.44
Carrier ^{1}H / ppm	0.449	0.427

TABLE S3. Experimental table for solid-state NMR acquisition and pulse program parameters used for the 3D-hNCAHA experiment on UL-Cp149.

**Experiment**	**3D hNCAHA**
^{1}H Field / MHz	1200
MAS frequency / kHz	160
Number of scans	32
Experimental time	30h24
t_1 increment	42
Spectral width (t_1) / ppm	38
Acquisition time (t_1) / ms	4.54
t_2 increment	72
Spectral width (t_2) / ppm	30
Acquisition time (t_2) / ms	3.93
t_3 increment	4096
Spectral width (t_3) / ppm	46.25
Acquisition time (t_3) / ms	36.86
^{1}H decoupling (swftppm) / kHz	10
^{13}C decoupling (WALTZ64) / kHz	5
^{15}N decoupling (WALTZ64) / kHz	5
MISSISSIPPI (120ms) / kHz	20
Interscan delay / s	2.1
**Transfer 1**	**HN dipolar**
^{1}H field / kHz	127
^{15}N field / kHz	28
Shape	Tangent ^{1}H
CP contact time / ms	2
**Transfer 2**	**NC dipolar**
^{13}C field / kHz	60
^{15}N field / kHz	97
Shape	Tangent ^{13}C
CP contact time / ms	20
**Transfer 3**	**CH dipolar**
^{1}H field / kHz	127
^{13}C field / kHz	28
Shape	Tangent ^{1}H
CP contact time / ms	0.5
Carrier ^{13}C / ppm	55
Carrier ^{13}N / ppm	115
Carrier ^{1}H / ppm	1.59

TABLE S4. Experimental table for solid-state NMR acquisition and pulse program parameters used for the 3D-hCCH and HcCH TOBSY experiments on UL-Cp149.

Experiment	3D hCCH TOBSY-WALTZ16	3D HcCH TOBSY-WALTZ16	3D hCCH TOBSY-C9
^{1}H Field / MHz	1200	1200	1200
MAS frequency / kHz	160	160	160
Number of scans	8	8	16
Experimental time	71h04	71h48	84h51
t_1 increment	118	110	86
Spectral width (t_1) / ppm	58	58	60
Acquisition time (t_1) / ms	3.36	3.65	2.37
t_2 increment	118	128	86
Spectral width (t_2) / ppm	58	58	60
Acquisition time (t_2) / ms	3.36	3.65	2.3
t_3 increment	4096	1400	4096
Spectral width (t_3) / ppm	46.25	46.25	46.25
Acquisition time (t_3) / ms	36.86	12.6	36.8
^{1}H decoupling (swftppm) / kHz	10	10	10
^{13}C decoupling (WALTZ64) / kHz	5	5	5
MISSISSIPPI (120ms) / kHz	20	20	20
z-filter / ms	10	10	10
Interscan delay / s	2.1	2.1	2.1
**Transfer 1**	**HC dipolar**	**HC dipolar**	**HC dipolar**
^{1}H field / kHz	131	131	118
^{13}C field / kHz	30	30	41
Shape	Tangent ^{1}H	Tangent ^{1}H	Tangent ^{1}H
CP contact time / ms	0.4	0.4	1.4
**Transfer 2**	**CC Jcoupling**	**CC Jcoupling**	**CC Jcoupling**
Shape	WALTZ16	WALTZ16	C9
^{13}C field / kHz	30	30	45.18
Mixing time /ms	11	11	15
**Transfer 3**	**CH dipolar**	**CH dipolar**	**CH dipolar**
^{1}H field / kHz	129	129	117
^{13}C field / kHz	30	30	41
Shape	Tangent ^{1}H	Tangent ^{1}H	Tangent ^{1}H
CP contact time / ms	0.5	0.5	0.5
Carrier ^{13}C / ppm	36.55	36.55	33
Carrier ^{1}H / ppm	4.8	4.8	4.8

TABLE S5. Total proton line width Δ^{tot} for *O*-phospho-L-serine measured at increasing MAS frequencies from 100 to 160 kHz and proton frequency of a) 850 MHz and b) 1200 MHz. Experimental errors (σ) are calculated as described in the Material and Methods section. The line width marked with a star are showing poor fits (see Fig. S7) due to the higher order terms in the AHT expansion.

a) Δ^{tot} for *O*-phospho-L-serine measured at 850 MHz proton frequency

MAS / kHz	Δ^{tot} (H') / Hz	Δ^{tot} (H_γ) / Hz	Δ^{tot} (H) / Hz	Δ^{tot} (H_α) / Hz	Δ^{tot} ($H_{\beta 2}$) / Hz	Δ^{tot} ($H_{\beta 3}$) / Hz
160	100.3 ± 0.7	109.1 ± 0.6	173.4 ± 1.2	128.8 ± 0.7	126.2 ± 1.8	120.2 ± 1.3
150	105 ± 2	113.7 ± 0.8	179 ± 2	135.2 ± 1.0	132 ± 3	129 ± 2
140	108 ± 2	117.6 ± 0.9	185 ± 2	145 ± 2	143 ± 3	139 ± 3
130	111 ± 2	121.4 ± 0.9	191 ± 2	130 ± 2	152 ± 3	159 ± 3
120	115 ± 2	127.3 ± 0.9	199 ± 2	173 ± 2	168 ± 3	167 ± 3
110	132.3 ± 0.1	145.1 ± 1.0	216 ± 2	280 ± 5	*	218 ± 6
100	126 ±2	141.2 ± 1.0	220 ± 2	*	204 ± 14	224 ± 6

b) Δ^{tot} for *O*-phospho-L-serine measured at 1200 MHz proton frequency

MAS / kHz	Δ^{tot} (H') / Hz	Δ^{tot} (H_γ) / Hz	Δ^{tot} (H) / Hz	Δ^{tot} (H_α) / Hz	Δ^{tot} ($H_{\beta 2}$) / Hz	Δ^{tot} ($H_{\beta 3}$) / Hz
160	130 ± 5	144 ± 5	183 ± 5	122 ± 7	128 ± 12	118 ± 7
150	129 ± 5	143 ± 5	187 ± 5	126 ± 7	133 ± 11	123 ± 7
140	129 ± 5	144 ± 5	192 ± 5	131 ± 7	142 ± 12	130 ± 7
130	135 ± 2	148 ± 4	198 ± 5	139 ± 8	152 ± 12	142 ± 7
120	134 ± 5	153 ± 4	206 ± 5	148 ± 8	164 ± 12	154 ± 8
110	138 ± 5	159 ± 4	216 ± 5	160 ± 8	180 ± 12	169 ± 8
100	143 ± 5	166 ± 4	227 ± 5	175 ± 8	199 ± 11	189 ± 8

TABLE S6 Bulk relaxation times of a) aliphatic protons and b) amide protons and ^{15}N of UL-Cp149. For the aliphatic protons, region A is defined between $\delta(^1H)$ = 2.75 – 5.9 ppm and corresponds to the Hα and region B is defined between $\delta(^1H)$ = 0 – 2.75 ppm and corresponds to the side-chain protons (C**H**$_2$ and C**H**$_3$).

a) Bulk relaxation times of aliphatic protons

Region A (field / MHz, MAS / kHz)	$T_1(H_\alpha)$ / s	$T'_2(H_\alpha)$ / ms	$T_{1\rho}(H_\alpha)$ / ms
850, 110	1.41 ± 0.01	3.0 ± 0.2	13.3 ± 0.3
1200, 110	1.75 ± 0.02	3.4 ± 0.1	13.8 ± 0.6
850, 160	1.51 ± 0.02	4.6 ± 0.1	18.5 ± 0.4
1200, 160	1.74 ± 0.03	5.0 ± 0.1	19.2 ± 0.6
Region B (field / MHz, MAS / kHz)	$T_1(H^{sc})$ / s	$T'_2(H^{sc})$ / ms	$T_{1\rho}(H^{sc})$ / ms
850, 110	1.37 ± 0.03	2.5 ± 0.1	13.1 ± 0.4
1200, 110	1.64 ± 0.03	3.0 ± 0.1	15.6 ± 0.8
850, 160	1.43 ± 0.03	3.8 ± 0.1	19.2 ± 0.7
1200, 160	1.53 ± 0.02	4.3 ± 0.1	21.0 ± 1.0

b) Bulk relaxation times of amide protons

(field / MHz, MAS / kHz)	$T_1(H_N)$ / s	$T'_2(H_N)$ / ms	$T_{1\rho}(H_N)$ / ms	$T'_2(^{15}N)$ / ms
850, 110	-	3.2 ± 0.1	19.8 ± 0.7	46.3 ± 1.9
1200, 110	2.06 ± 0.08	3.3 ± 0.8	14.9 ± 0.8	37 ± 2
850, 160	1.55 ± 0.09	4.9 ± 0.1	28.4 ± 0.7	56.1 ± 2.9
1200, 160	-	4.9 ± 0.8	20.7 ± 0.7	36.5 ± 0.5

TABLE S7 Site-specific $T_2'(^1H^{ali})$ times and $\Delta^{homo}(^1H^{ali}) = 1/(T'_2(^1H^{ali}) \cdot \pi)$ for the 51 residues (labeled in blue in Fig. S8) of UL-Cp149 at 160 kHz and 110 kHz MAS frequency and a proton frequency of 1200 MHz, obtained from the mono-exponential fits in Fig. S4 and 5. The experimental standard deviations (σ) are calculated as described in the Material and Methods section. The T_2' standard deviations (σ) marked with a star are higher than the T_2' value, indicating a poor fit due to low SNR data for the corresponding residues as shown in Fig. S4 and S5.

	110 kHz MAS				160 kHz MAS			
Residue	T_2' / ms	$\sigma(T_2')$ / ms	Δ^{homo}/ Hz	$\sigma(\Delta^{homo})$/ Hz	T_2' / ms	$\sigma(T_2')$ / ms	Δ^{homo}/ Hz	$\sigma(\Delta^{homo})$/ Hz
34AlaHb	4,2	1,2	75,1	39,1	8,1	1,7	39,1	7,4
80AlaHb	7,2	2,1	43,9	24,0	13,2	3,7	24,0	5,4
41AlaHb	4,9	1,0	64,4	43,9	7,3	0,7	43,9	4,3
33ThrHg2	2,6	0,3	123,4	57,4	5,5	0,6	57,4	6,3
70ThrHg2	7,2	1,1	43,9	11,4	27,8	*	11,4	4,9
67ThrHg2	8,3	*	38,5	14,4	22,1	6,0	14,4	2,9
68LeuHda	3,3	0,9	96,2	74,1	4,3	0,5	74,1	9,4
28ArgHba	3,1	0,2	103,3	69,4	4,6	0,4	69,4	6,6
68LeuHg<br>79ProHba	2,5	0,2	127,5	72,7	4,4	0,2	72,7	2,8
25ProHga	2,7	0,3	116,4	76,8	4,1	0,3	76,8	5,6
14GluHba	3,4	0,5	94,4	65,5	4,9	0,3	65,5	4,6
74ValHb	4,1	0,4	76,8	48,4	6,6	0,6	48,4	4,8
96LysHea	7,7	0,7	41,6	36,6	8,7	0,7	36,6	3,2
112ArgHda	4,3	1,5	74,1	51,7	6,2	*	51,7	9,5
10GlyHaa	3,4	1,5	93,6	45,7	7,0	2,3	45,7	11,8
73GlyHaa	14,4	*	22,1	81,3	3,9	0,9	81,3	15,6
63GlyHaa	5,2	*	61,1	54,6	5,8	0,9	54,6	8,5
94GlyHaa	2,6	*	122,4	41,8	7,6	1,3	41,8	7,2
79ProHda	1,2	0,3	256,7	89,4	3,6	0,7	89,4	16,4
78AspHa	7,3	10,5	43,4	52,1	6,1	1,5	52,1	11,3
22AspHa	5,8	*	54,4	20,7	15,3	24,6	20,7	7,0
2AspHa	6,0	1,4	52,8	27,4	11,6	2,7	27,4	5,6
46GluHa	4,6	*	68,9	34,6	9,2	2,6	34,6	8,1
36AlaHa	35,1	*	9,1	21,7	14,6	1,3	21,7	2,0
41AlaHa	5,8	2,3	54,9	39,8	8,0	1,2	39,8	5,4
76LeuHa	4,9	*	65,4	53,4	6,0	1,9	53,4	12,5
90AsnHa	6,1	1,2	51,8	57,5	5,5	0,7	57,5	7,4
108LeuHa	4,5	1,2	70,2	36,4	8,7	1,0	36,4	3,9
84LeuHa	4,4	0,6	72,4	35,4	9,0	1,3	35,4	4,4
110PheHa	3,7	1,1	85,7	38,9	8,2	1,6	38,9	6,5
82ArgHa	2,8	0,2	114,8	57,1	5,6	0,5	57,1	5,1
14GluHa	4,5	0,3	70,4	51,2	6,2	0,6	51,2	4,5
7LysHa	4,9	1,7	64,8	53,1	6,0	1,1	53,1	8,7
56ArgHa	4,3	0,5	74,1	64,6	4,9	0,4	64,6	5,3
113GluHa	2,4	0,4	132,3	66,8	4,8	0,8	66,8	10,8
39ArgHa	2,8	0,2	115,3	61,1	5,2	*	61,1	4,0
50ProHa	3,6	1,4	89,4	21,3	14,9	*	21,3	10,4
91ThrHa	3,8	0,8	84,5	69,1	4,6	1,0	69,1	12,2
130ProHa	3,0	0,7	105,7	47,5	6,7	1,2	47,5	7,9
79ProHa	2,7	0,3	117,9	56,1	5,7	0,3	56,1	2,7
48CysHa	3,2	*	100,7	54,6	5,8	1,2	54,6	10,1
105IleHa	4,0	0,6	80,5	30,5	10,4	21,7	30,5	11,1
59IleHa	3,4	0,4	94,8	57,3	5,6	0,5	57,3	5,3

70ThrHa	6,1	1,0	52,5	50,1	6,4	0,8	50,1	7,0
67ThrHa	6,4	1,5	49,5	43,5	7,3	1,0	43,5	5,9
85ValHa	4,0	1,1	78,7	46,2	6,9	0,9	46,2	6,3
115ValHa	3,4	0,7	93,5	79,7	4,0	0,3	79,7	5,1
27ValHa	2,8	0,5	113,6	45,7	7,0	3,4	45,7	13,1
86ValHa	9,3	*	34,1	66,4	4,8	0,7	66,4	8,1
67ThrHb	4,6	2,9	69,5	49,0	6,5	0,6	49,0	4,3
70ThrHb	6,0	1,0	53,4	50,3	6,3	0,7	50,3	5,0
